# Host Range, Biology, and Species Specificity of Seven-Segmented Influenza Viruses—A Comparative Review on Influenza C and D

**DOI:** 10.3390/pathogens10121583

**Published:** 2021-12-05

**Authors:** Chithra C. Sreenivasan, Zizhang Sheng, Dan Wang, Feng Li

**Affiliations:** 1 Maxwell H. Gluck Equine Research Center, University of Kentucky, Lexington, KY 40546, USA; chithra.sreenivasan@uky.edu (C.C.S.); dan.wang@uky.edu (D.W.); 2 Aaron Diamond AIDS Research Center, Vagelos College of Physicians and Surgeons, Columbia University, New York, NY 10032, USA; zs2248@columbia.edu

**Keywords:** seven-segmented, eight-segmented, influenza C, influenza D, host range, species specificity, structure, biology, receptor 9, transmission

## Abstract

Other than genome structure, influenza C (ICV), and D (IDV) viruses with seven-segmented genomes are biologically different from the eight-segmented influenza A (IAV), and B (IBV) viruses concerning the presence of hemagglutinin–esterase fusion protein, which combines the function of hemagglutinin and neuraminidase responsible for receptor-binding, fusion, and receptor-destroying enzymatic activities, respectively. Whereas ICV with humans as primary hosts emerged nearly 74 years ago, IDV, a distant relative of ICV, was isolated in 2011, with bovines as the primary host. Despite its initial emergence in swine, IDV has turned out to be a transboundary bovine pathogen and a broader host range, similar to influenza A viruses (IAV). The receptor specificities of ICV and IDV determine the host range and the species specificity. The recent findings of the presence of the IDV genome in the human respiratory sample, and high traffic human environments indicate its public health significance. Conversely, the presence of ICV in pigs and cattle also raises the possibility of gene segment interactions/virus reassortment between ICV and IDV where these viruses co-exist. This review is a holistic approach to discuss the ecology of seven-segmented influenza viruses by focusing on what is known so far on the host range, seroepidemiology, biology, receptor, phylodynamics, species specificity, and cross-species transmission of the ICV and IDV.

## 1. Introduction

Influenza viruses, first documented in the late 16th century [1], have evolved into four major types—alphainfluenza virus (influenza A), betainfluenza (influenza B), gammainfluenza (influenza C), and deltainfluenza (influenza D) viruses. Besides Isavirus, Quaranja, and Thogoto viruses in the Orthomyxoviridae family, the delta influenza (influenza D/IDV) was added as the fourth genus to the pre-existing three genera—alpha, beta, and gamma influenza [2]. Within each type, there are subtypes, clades, lineages, and sub-lineages. The advantage of being a quasispecies virus, coupled with its extremely rapid evolution dynamics largely via genetic drift, and reassortment events, influenza viruses can generate biologically significant genomic variants in multiple mammalian host species. However, the superior replication fitness, ability to surpass immune selection pressure, and invasion of adaptive landscapes determine the sustainability and ecology of the viruses. Influenza viruses have a very broad host range, and the species specificity of influenza viruses largely depends on the host-specific cellular and biochemical components and their potential to overcome these bottlenecks for adaptation and sustenance.

Structurally, IAV and IBV have eight-segmented genomes, whereas ICV and IDV have seven-segmented genomes. Other than humans, IAV affects diverse host species such as pigs, dogs, horses, cats, minks, whales, and seals, and poultry. Reports of IBV in pigs, seals, dogs, and horses were also occasionally documented [3,4,5,6,7,8,9]. However, domestic ruminant animals of bovine, ovine, and caprine species were unaffected, and hence these species are considered refractory to eight-segmented influenza A and B viruses, irrespective of a few isolated reports on isolation/seroprevalence of human/swine IAV [10,11] and IBV from cattle in the past [12]. Despite the co-circulation of several influenza strains and the growing host range of IAV that included most of the terrestrial/marine mammals over the last century, influenza A virus could not breach the avian–bovine or human–bovine, species barriers, and hence it can be inferred that influenza A evolution witnessed an unfavorable stance in bovine species. Interestingly, the ruminant species were quite susceptible to the seven-segmented influenza viruses, ICV and IDV, of which cattle have been considered as the primary reservoir for influenza D viruses. This review describes the ecology of ICV and IDV by discussing the cross-species transmission, host range, phylodynamics, virus biology, species specificity, and receptor preferences.

## 2. Cross-Species Transmission of Influenza C and D Viruses

The first case of ICV in humans was reported in 1947 from New York, USA [13], from the throat washings of asymptomatic and symptomatic individuals sampled during the late Fall–early Winter; however, reports of serological evidence indicate that ICV was prevalent even before 1947 [14]. Increasing cases of influenza viruses were reported from North America and Europe, especially in the child population [15,16,17,18]. As we analyze the ecology of influenza C, several questions need to be addressed. What triggered influenza C, which is primarily a human pathogen to spillover into pigs? The first animal-associated ICV was reported in pigs in the 1980s in Beijing, China. From there, it took another couple of years to spill over to canines. When IDV was discovered in 2011, it was initially thought that it was a case of ICV. However, the IDV genome, though seven segmented and shares 50% sequence identity to ICV, demonstrated certain salient features unique to its pathogenesis, tissue tropism, and host range. IBV and ICV are the two influenza viruses with humans as the primary host. Influenza B viruses were discovered in 1940, a little before ICV was isolated [19]. Over the years, IBV spilled over to cause natural infections in limited species such as seals and lately in pigs. On the other hand, influenza C viruses crossed the species barrier from humans to infect dogs, pigs, camelids, and horses as evidenced by the seroprevalence, virus isolation, and natural infection studies [20,21]. Interestingly, ICV also can cause natural infections in bovines and the bovine origin ICV strains shared high sequence identity to human influenza viruses indicative of reverse zoonosis [22]. Hence, it is worthwhile to study the host or viral factors that favored ICV over IBV, to spill over to other species.

Compared with ICV, IDV came to our attention only in recent years because it was detected ten years ago, with bovines as the primary host. However, the infection landscape and host range of IDV widened over these years and the virus spilled over periodically to pigs, sheep, goats, camels, and horses. Influenza D virus originally isolated from pigs suffering from respiratory illness turned out to be a transboundary pathogen in cattle populations worldwide. A seroepidemiological study in Italy over the years 2005–2017 revealed serological evidence of IDV in 2005 [23] before it was discovered in 2011 in the USA. Moreover, the presence of IDV in the nasal wash of a pig farm worker also suggests that the IDV can infect humans [24]. IDV has also been detected in human environments such as hospitals, airports, and emergency rooms [25]. Furthermore, serosurveillance studies have revealed that IDV antibodies were seen in occupational workers [26]. Despite showing no serological evidence of IDV in poultry [27], molecular evidence of IDV has been found lately in that 14% of the bioaerosol samples collected from five poultry farms in Malaysia tested positive for IDV [28]. Therefore, compared with ICV, IDV is a zoonotic agent, and it is still unknown whether this can cause any sub-clinical/clinical infections or whether the cross-reactive ICV antibodies in humans can combat infection.

**Figure 1:**
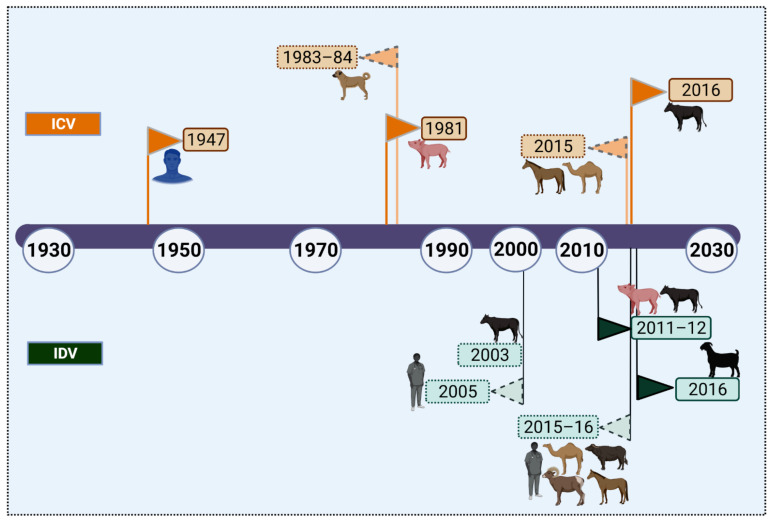
Timeline of natural infections of the influenza C and D viruses based on the chronological order of occurrence. The key events of ICV (orange flags) and IDV (green flags) were described on the top and bottom of the year bar, respectively. The right-facing flags (dark orange and dark green) represent the events of virus isolation and the left-facing flags with dotted lines (light orange and light green) denote the events of seroprevalence. Following the first detection of IDV in 2011–2012, serological studies using archived bovine and human sera conducted in the USA and Italy found evidence of IDV circulation in 2003–2005, which is also shown in the figure. The year of the event is also marked next to the flag and the species involved is also illustrated. The graphical illustration was created with BioRender.com, accessed 30 November 2021.

The absence of adaptive evolution is the main hurdle for the establishment and emergence of influenza in a species. This, in turn, could be attributed to host-specific genetic and cellular factors interfering with viral attachment, receptor binding, fusion, or release [29,30,31], leading to sub-optimal gene constellation/gene reassortments necessary for establishing the niche for sustained interspecies transmission. A timeline of the natural infection events of ICV and IDV in the chronological order of occurrence is illustrated in Figure 1. In the case of ICV, the timeline of its index case with successful virus isolation in humans (1947) to the first case in pigs (1981–1982), then to the first reported natural infection in dogs (1983-1984), and to the first case in bovines (2016) show that adaptive evolution of ICV is quite slow. On the other hand, IDV, though officially discovered in 2011 has been evidently circulating in the USA and Italy since at least 2003–2005 (Figure 1), based on the serological studies conducted in bovines and humans [23,32,33]. While it is unknown whether IDV was prevalent before 2003, serological screening of 2083 samples spanning 1991–2015 collected from swine, cattle, dromedary camels, and small ruminants of North and West Africa indicated that IDV started circulating only by 2012 [32]. Nevertheless, IDV has been found to have a broader host spectrum with evidence of natural infection in humans, pigs, small ruminants (sheep, goat), camel, and horses, which indicates that IDV can spill over to other species easily compared with its distant relative, ICV. With the advent of molecular and next-generation sequencing methods, pathogen discovery is easier now which can generate rapid, precise, and timely information as with the identification of IDV genome in human environments such as hospitals, airports, farms, etc. Though ICV exhibits a slow evolution, it has exceptional long-term stability in vivo [33]. Furthermore, the superior environmental stability of IDV compared to ICV [34] together with its ability to interact with diverse glycans and its broad host tropism could lead to gene segment interactions between these two similar structured viruses with shared receptor preferences, and hence the emergence of viable replicative competent ICV-IDV reassortants cannot be dismissed, even though the in vitro reassortment experiment failed to isolate a viable reassortant.

## 3. Ecology of Seven-Segmented Influenza Viruses

**Figure 2:**
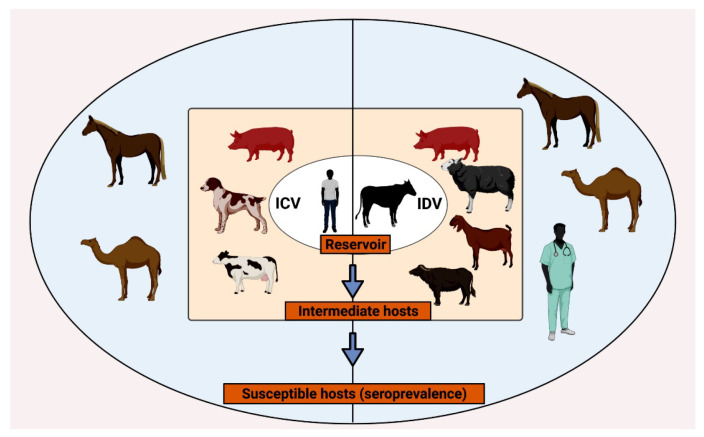
The host ecology of ICV and IDV. The principal hosts/reservoir animals of ICV (humans) and IDV (bovines) have been described in the innermost (white) oval space, followed by the intermediate hosts in the (light orange) middle space based on the natural infection, ICV: swine, canine, bovines; IDV: swine, bovines, caprine, bubaline, etc. The susceptible hosts are shown based on serological information in the outermost (light blue) space. Equines and camelids are susceptible hosts for both ICV and IDV, whereas humans are susceptible to IDV. The graphic image was created with BioRender.com, accessed 30 October 2021.

The host ecology of the ICV and IDV has been described in Figure 2. The reservoirs of the ICV and IDV are humans and bovines, respectively. The presence of the ICV genome has been detected in pigs [35,36,37], dogs [5,21,37,38], and bovines [22,38] and can be considered as the incidental or intermediate hosts of ICV, whereas pigs, goats, sheep, and buffaloes were included as the intermediate hosts of IDV [27,39,40,41,42,43,44,45]. The other species which are susceptible and were reported to be naturally infected as indicated by the seroprevalence data include horses [46] and camelids [32] for both ICV and IDV. In the case of IDV, serological evidence of IDV has been reported in occupational workers in the US [26] and Malaysia (Figure 2). Natural and experimental hosts of ICV and IDV have been described in this section.

### 3.1. Natural Hosts of Influenza C and D Viruses

#### 3.1.1. Human

Humans are the primary natural hosts of ICV, and it causes mainly upper respiratory tract infections. The first clinical case of ICV related respiratory infection was reported in 1947, an epidemic (March–April) in a vocational school in New York. As part of the clinical survey, throat washings were collected from schoolboys (17–21 years of age) with and without upper respiratory symptoms from November 1946 to April 1948 [13]. The symptoms manifested were milder with upper respiratory signs, mild headache, and myalgia. Furthermore, the virus was isolated in 1949, by two groups of research workers, and named as the 1233 strain by the Taylor group [47] and the J.J strain by Francis et al. [48]. Because of its distinct antigenic difference from influenza A and B types, both the strains were later classified under influenza C. Later on, after studying the hemagglutinin esterase fusion protein (HEF), and the O-acetyl esterase activity of HEF compared with the neuraminidase activity of IAV and IBV, it was officially classified as a separate genus under *Orthomyxoviridae* [49]. 

ICV causes acute respiratory illness in children with a myriad of symptoms such as fever, nasal discharge, conjunctivitis, cough, asthenia, infrequent breathing problems, vomiting, and diarrhea. In adults, most of the infections are mild. Analyses of infection dynamics of ICV changed lately and it was linked to community acquired pneumonia and is associated with bronchitis and pneumonia [49,50,51], and co-infections involving different bacteria and viruses often complicate the real clinical significance of ICV-related respiratory diseases, particularly in children [52,53]. The advent of metagenomic RNA sequencing has detected the co-infections of human coronavirus HKU-1, ICV, and human PIV-2 in neurogenic pulmonary edema in a 22-month-old baby [53].

ICV co-infections with influenza A and B types also occur and were reported as early as 1951 [17,54]. During 1950–1960, ICV infections were reported from different parts of Eurasia [15,17,54,55,56,57,58,59,60] in children and also in adults aged 45–69 years [17]. Similar to A and B influenza types, fatal cases of ICV infections complicated with *Staphylococcus aureus* in adults were also reported, which is also the first reported ICV case from the UK [16]. 

Sero-epidemiological studies have shown that ICV antibodies were present in all humans worldwide, especially with a high seropositivity rate in children less than 5 years of age [61]. The seroprevalence of 98–100% ICV in the 11–25 years age group, compared to 64% in children less than 5 years of age, indicates that ICV is a very common pathogen during childhood [62,63]. Seroprevalence of ICV in humans and other susceptible species is given in Table 1. Sero-epidemiological studies are needed to determine the major antigenic groups in circulation, although cross-reacting antibodies are very common among different ICV strains. A longitudinal study conducted in Sendai, Japan from 2011–2016 revealed an ICV infection rate of 17.5%, and C/Sao Paulo was the predominant lineage, with co-circulation of C/Kanagawa in between [64]. A similar surveillance study in Hong Kong SAR, China, over two winter periods (2015–2016, 2017–2018) has also shown Sao Paulo and Kanagawa lineage, represented by C/Kanagawa/1/76 and C/São Paulo/378/82 as the predominant viruses in circulation [65].

Although the zoonotic potential of IDV is not yet established, it is quite surprising to note the 95% seroprevalence against D/Bovine/Kansas/1-35/2010 virus in occupational workers in Florida [26]. Even though this is an alarmingly high seroprevalence in occupational workers, no other serosurveillance studies have been conducted on occupational people in this area or elsewhere so far. Serological evidence of IDV was found in the Italian human population in samples dated back to 2005, indicating that the IDV has been in circulation for a while before its official discovery [23]. However, a molecular surveillance study conducted using 3300 human respiratory samples collected during 2006–2008 in Scotland did not detect any IDV, where ICV was present in 0.2% of the samples from persons <2 years >45 years old [73]. In vitro studies showing the binding capacity of IDV to human respiratory tissues as well as its replication competency in human continuous cell lines and primary cells suggest that IDV can replicate in human respiratory tissues [74,75]. IDV has been detected along with other respiratory and enteric pathogens from the nasal wash samples of farmworkers in Malaysia recently [24] and also from human premises [25]. Clearly, this area of public health significance demands further studies from different geographical locations as more and more evidence is needed for uncovering the zoonotic potential of this virus.

#### 3.1.2. Bovines

##### Influenza D Virus

Bovines are the primary hosts for IDV. IDV has been reported from China, Japan, Italy, France, Ireland, Argentina, Morocco, Benin, Turkey, Kenya, and Togo [32,39,41,45,76,77,78,79,80,81]. Although IDV was originally isolated from swine showing influenza-like respiratory illness in the USA, serological screening has revealed higher seroprevalence against IDV in bovine herds compared with the swine, with geometric mean antibody titer (GMT) of >40) [82]. Respiratory samples collected from the bovines also tested positive for IDV by RT-qPCR and several bovine IDV strains were isolated from different parts of North America [82]. Similar to humans, a retrospective serological study using cattle sera collected in 2003–2004 indicated that IDV seropositive adult animals were present back then and 98% of the newborn calves carried IDV-specific maternal antibodies at high levels, which indicates that IDV was present in the US before 2011 [83]. Serological screening in archived samples from Mississippi beef cattle revealed that IDV has been circulating since 2004 [84].

Following the initial reports of IDV from the USA, several reports of IDV have emerged from other parts of the world such as France, China, Japan, and Italy, which shows its intercontinental transmission [44,49,82]. A nationwide survey conducted on the serum samples of bovine herds collected from different parts of Japan from 2010 to 2016 also showed seropositivity of 30.5% in cattle, irrespective of the breed [41]. IDV antibodies were also detected in 80.2% (361/450) of the cattle sampled in Luxembourg during 2012–2016, where all the samples were derived from animals more than 6 months of age, dismissing the possibility of maternal antibodies. In Luxembourg, about 88% of the beef cattle showed seropositivity and the sampled animals were exclusively born in Luxembourg, and also from neighboring countries such as Germany, France, Belgium, and Italy. The herd prevalence in in-land and near-border cattle herds were similar, indicating the possibility of IDV transmission from the cattle population of France and Italy while cross-border grazing [43]. Other than cattle, buffaloes also tested positive for IDV in China [45].

##### Influenza D Linked to Bovine Respiratory Disease

Bovine seroepidemiological studies of IDV revealed seroprevalence in 87.5% of the U.S bovine herds, even though clinical screening of swine and cattle nasal samples revealed 1% of the samples were positive by qRT-PCR in the USA. Under natural conditions, IDV alone is not a disease-causing agent, but the rate of IDV detection in sick compared with healthy cattle was more, hence it was believed that IDV can cause respiratory disease in bovines. The morbidity rate of bovine respiratory disease complex (BRDC) is about 70–80% in the USA that is caused by a single or combination of factors such as environmental, host, managemental, and infectious factors, posing a significant economic burden affecting the cattle industry in North America [85]. About 4% and 3–7% of BRDC diagnostic submissions were positive for ICV and IDV by qRT-PCR. IDV and bovine coronavirus (BCV) are associated with 5/10 positive BRDC samples, suggesting a primary role for IDV in BRDC [22,86]. Another striking feature is that ICV, BCV, and IDV use 9-*O*-acetylated sialic acids (Neu5,9Ac2) as the cellular receptor for virus entry [86]. A metagenomic virome study conducted in nasal swab samples from the feedlot cattle detected IDV in 17% of the samples derived from symptomatic animals and found a significant association of IDV in diseased animals compared with other viruses associated with BRDC [87]. IDV RNA was also present in asymptomatic animals. These findings coupled with the frequent isolation of IDV from bovines across the globe suggested that cattle could be the reservoir for IDV [87]. A higher incidence of IDV (29.1%) was also reported in calves with BRDC compared with healthy (2.4%) animals [84]. Another study of PCR screening of nasopharyngeal swabs from swiss veal calves also revealed 53.5% of bovine coronavirus and 4.1% IDV with no ICV detection [88]. A nasal virome study in Western Canada involving 310 deep nasal swabs from cattle with known BRDC outcomes has revealed that the association of IDV is the same with 3.55% prevalence in animals with (11/310) and without BRDC (11/310) [89]. Nasal swabs/tracheal washes in beef cattle revealed a 6.9% prevalence, whereas, in lung samples, only 0.77% was noted [90,91]. Furthermore, 3/76 samples from 12 cattle herds in Turkey (3 weeks–6 months) affected with respiratory disease tested positive for IDV and phylogenetic analyses of HEF protein showed that these Turkish strains shared 95% sequence identity to the European and US sequences, indicating the intercontinental transmission of IDV [92]. These pieces of evidence from different parts of the world have strongly established a synergistic role of IDV in the development of the BRDC in bovines.

Global seroepidemiology of the influenza D virus showed seroprevalence in ruminants that include dairy cattle, buffaloes, sheep, and goats (Table 2). Lately, more swine IDV viruses have been isolated from different parts of Eurasia [44,49,81]. Table 2 showed the year-wise seroprevalence observed for different species from North America and Eurasia [27,44,49,81,82].

##### Influenza C Virus

Even though seroepidemiological studies conducted in the past have demonstrated the presence of antibodies against influenza C in bovines (0.6%) around the late 1970s [4], influenza C prevalence in cattle or any other ruminant population was not reported until 2016. Lately, seroepidemiological studies have demonstrated that both ICV and IDV are prevalent in the bovine populations [32]. ICV has also been detected in 64/1525 (4.2%) from the nasal swabs and lung specimens of the bovines suffering acute respiratory disease collected during October 2016–January 2018 by RT-qPCR [22], which suggests that both ICV and IDV can be linked to BRDC [22,95]. Another study using a larger cattle cohort from Colorado, Washington, and New Mexico states of the USA (*n* = 599), employing viral RNA screening by RT-PCR and disease scoring for respiratory symptoms revealed that cattle with higher IDV and ICV loads also harbored other co-infecting viruses (BcoV, PI3, BoHV-1, BVDV, and BRSV) compared with uninfected cattle and suggested significant association of the ICV/IDV to BRDC [38]. This study also found that ICV is more prevalent than IDV in non-BRD cattle, which raises the question as to whether humans are the only primary reservoir of ICV [38]. Furthermore, sequencing of a 590-bp fragment of the matrix gene from 12 ICV-positive bovine samples, showed a high nucleotide sequence identity of approximately 98% between strains and 95% sequence identity to the human ICV strains, which clearly indicates the zoonotic potential and possibly a bidirectional transmission of ICV at the human-cattle interface [22]. Of the twelve ICV strong positive samples, four samples also harbored IDV, as demonstrated by the qRT-PCR. The complete genome sequence of C/bovine/Montana/12/2016 is available in the NCBI database [95]. While it remains unknown whether ICV and IDV undergo reassortment in bovines to cause disease and facilitate cross-species transmission, the involvement of IDV in the BRD complex has been established beyond doubt. 

#### 3.1.3. Small Ruminants

Quast et al. found that small ruminants (sheep and goat) can become naturally infected with IDV as evidenced by the presence of antibodies. About 648 sera (collected in 2014) from the small ruminants from different locations in the US and Canada were included in the study and found that 13.5% (17/126) and 13.3% (2/15) of the sheep and goat farms tested positive against IDV. Approximately 5.2% (29/557) of sheep sera and 8.8% (8/91) of the goat sera harbored IDV antibodies, indicating the natural susceptibility of these two species to IDV [27]. Serological evidence of IDV in goats (2.2%) and sheep (1.4%) were also reported from Togo, Africa (samples collected in 2013). Evidence of IDV in goats was reported from Guangdong Province of Southern China [45]. No evidence of ICV has been found in small ruminants yet.

#### 3.1.4. Pigs

Pigs are the first animal species reported to have seasonal ICV activity in China. Fifteen ICV strains were isolated from apparently healthy pigs at a slaughterhouse in Beijing from January–December 1981 and experimental infection of porcine and human ICV strains in experimental pigs also showed that ICV can remain infectious for 25 days and can be transmitted by contact [35]. The porcine ICV isolates also possessed seven RNA segments of the same size as human ICV isolates [37].

Influenza D was first isolated from an ailing swine (D/swine/Oklahoma/1334/2011); however, the clinical detection rate of IDV in swine nasal samples was low despite high seroprevalence [41,48,96]. Influenza D was detected only in 2/2862 nasal swabs collected from 102 swine exhibitions [97]. However, IDV infections from pigs were reported more from Italy and China, and more swine IDV strains were isolated [77,78]. Clinical swine lung samples tested in China for IDV showed a positivity rate of 28.9% [45]. Luxembourg swine herds also showed a gradual increase in seroprevalence over time, with 20.7% of swine herds containing seropositive animals [43]. A recent study evaluated the seroprevalence of IDV in feral swine and found that 19.1% of the 256 tested sera sampled during 2012–2013 from the four US states were positive for IDV. Furthermore, 41 (42.7%) of 96 archived IAV seropositive feral swine samples were positive for IDV, which originated from 16 US states during 2010–2013 [96]. However, IDV was not detected in Vietnam from diverse samples collected from five swine farms in 2019–2020, which involved bio-aerosol samples, fecal samples, nasal washes from farmworkers, and oral secretions from swine [98]. A screening of the 86 bioaerosol samples in a swine farm, North Carolina indicated that only 1/86 samples were positive for IDV other than PCV2, PCV3, and IAV [99]. Pigs are considered as the mixing vessels of influenza, and the susceptibility of pigs to all four types of influenza is, therefore, alarming and needs further investigation.

#### 3.1.5. Dogs

Dogs were naturally infected with ICV as suggested by the seropositive samples in Japan and France. In France, around 32% (*n* = 134) of the tested cases were positive for ICV antibodies, although there was no significant age-dependent correlation [20]. A molecular epidemiological study of oropharyngeal swabs conducted in New Zealand from 116 dogs (56 canine infectious respiratory disease syndrome (CIRDS)-affected dogs and 60 healthy dogs) revealed influenza C virus along with canine respiratory coronavirus (CRCoV), canine herpesvirus-1 (CHV-1), canine picornavirus, and canine pneumovirus (CnPnV) in CIRDS dogs [100]. No such studies have been carried out for IDV in dogs; however, it is worth investigating as evidence of IDV has been detected in humans, and dogs are companion animals. 

#### 3.1.6. Horses

The seroprevalence of horses in the midwestern states of the United States against the two lineages of IDV has been studied and found that 12% (44/364) of samples were seropositive against D/OK lineage virus, whereas 11% (39/364) were positive for D/660 lineage virus. Furthermore, 11% of the samples were seropositive against ICV, C/Johannesburg/1/66 [46] which shows that horses are susceptible hosts to both ICV and IDV.

#### 3.1.7. Camels

Recently a very high IDV seroprevalence rate has been reported in camelids [32]. Dromedary camels showed high seroprevalence against D/bovine/Nebraska/9-5/2012 in Kenya, as demonstrated by the HI assay [32]. This study demonstrated a high IDV prevalence rate of (99–100%) in camelids with a GMT of 150, whereas the ICV seroprevalence rate was 94% with a GMT of 38 suggesting cross reactivity. Another study in dromedary camels in Ethiopia using D/swine/Oklahoma/1334/2011 (D-OK lineage; D/OK) (1), D/bovine/Nebraska/9-5/2013 (D/660-lineage; D/NE) (6), and D/bovine/Yamagata/10710/2016 (D/Japan-lineage; D/Yamagata) showed 55.2% seroprevalence with high seropositivity against D/bovine/Yamagata/10710/2016 and no cross-reactivity between ICV and IDV [42]. It is interesting that Bactrian camels, however, did not harbor any IDV/ICV antibodies [42]. The susceptibility of dromedary over Bactrian camels is an example of species and receptor specificities and holds scope for investigation. This suggests that the camel could be a natural host for both ICV and IDV [32].

#### 3.1.8. Poultry

Serosurveillance of poultry sera (chicken and turkey) reported no evidence of IDV in poultry [27]. However, the IDV genome was reported from a bioaerosol surveillance study in a Malaysian poultry farm indicating that poultry could be a potential host for IDV [28]. No such reports on ICV have been documented yet. 

### 3.2. Experimental Hosts

#### 3.2.1. Influenza C

Embryonated chicken eggs were used as early as the 1950s for the isolation of influenza C viruses and for studying the ICV and bacterial co-infection [101,102]. Whereas the initial isolation of the 1233 strain (Taylor) induced some pathological lung lesions in the embryo, the J.J strain did not cause any degenerative or inflammatory changes in the embryo [13,103]. Immune sera were prepared by intranasal inoculation of ferrets and hamsters and by the intraperitoneal inoculation of rabbits. However, it was reported that repeated attempts to infect mice have failed [13]. It was also reported that active proliferation of bacteria in chicken embryonic cultures hindered ICV replication, showing that bacteria can produce substances detrimental to the virus propagation [104]. The differential detection of ICV from IAV/IBV was a difficult task before the molecular detection methods were in place, and it was recommended to incubate the chicken embryonated eggs for 5 days instead of 2–3 days for A and B viruses. The differential diagnosis of ICV was based on the ability of the ICV in the harvested amniotic fluid–chicken RBC mixture to undergo elution at room temperature [105].

Other experimental animals used for ICV studies include dogs, pigs, and rats. Whereas other disease outcomes remained variable, nasal shedding with seroconversion was observed in all these hosts. Pigs were used as experimental hosts to study swine and human ICV strains and both strains demonstrated nasal shedding in direct inoculated and contact-infected animals until 11 days post-infection [35]. Beagle dogs inoculated intranasally with ICV showed mild rhinorrhea and cough and showed HI titers 3 weeks after infection [106]. However, mongrel dogs infected with Ann Arbor/1/50 strain of ICV caused nasal discharge, swelling of the eyelids, lacrimation within 1–4 days, high HI titers, and nasal shedding. Most of the infected dogs showed symptoms for 10 days, a clinical characteristic comparable to human cases [107]. To check the airway hyperresponsiveness, dogs were exposed to acetylcholine and infected with influenza C, and then compared with non-infected control dogs, and histology of the tissues was studied. Epithelial damage, inflammation, and luminal mast cell proliferative changes were more common in the ICV-induced hyper-reactive dogs [108]. 

The intranasal inoculation of four-week-old rats with C/Ann Arbor/1/50 of ICV showed no clinical signs; however, it caused nasal shedding for 10–20 days, and seroconversion [109]. The viral shedding peaked at 24 hpi, and stayed until 5 dpi [109]. Administration of convalescent sera before ICV infection rendered complete resistance to lung infection in rats [110]. Interestingly, the ICV genome was detected in the chicken lung by RT-PCR until 53 dpi, indicating the long-term stability of the ICV genome [33]. ICV also caused deformities in cerebellar Purkinje cell arrangement and also abnormal dendritic arborization patterns in ICV-infected chicken embryos compared with the uninfected control [111]. Earlier, human volunteers were used for studying the pathogenesis study of C/Johannesburg/1/66 (intranasal inoculation) and the clinical signs included malaise, headache, pyrexia, cough, and sore throat. The volunteers also exhibited a rise in hemagglutination inhibition and complement fixation titers in volunteers with and without colds [112]. 

#### 3.2.2. Influenza D

Calves were used for experimental studies of IDV (detailed in the pathobiology in principal host species). IDV can be experimentally infected in ferrets, pigs, and guinea pigs. Influenza D infection in pigs has shown that IDV could replicate in the respiratory tract and also can be transmitted to other animals by contact [113]. Intranasal inoculation of IDV in swine did not manifest any clinical signs but caused nasal virus shedding and transmitted the virus by direct contact. Histopathological examination of the lung did not show any typical influenza-like lesions in the swine [82]. Airborne transmission in pigs did not result in virus shedding by the sentinel animals; however, one of the sentinel animals showed seroconversion [82].

Intranasal inoculation of IDV in ferrets resulted in nasal shedding of the virus by 3 days post-infection (dpi) and also transmitted disease via contact by 7 dpi [114]. In guinea pigs, IDV demonstrated productive replication in the upper and lower respiratory tract. Even though the guinea pigs did not show any clinical signs, these animals were able to shed virus in the nasal washes and also demonstrated the highest viral titer in the lungs in both direct inoculated as well as in the contact sentinel animal. Histopathological lesions were confined more to the lungs with more diffused neutrophilic infiltrations in the lung alveolar spaces, lymphoblastic, and plasmacytic cell cuffing around the airways, and denudation of bronchial epithelium and presence of exudate and dead cells in the bronchial lumen [115]. Immunohistochemical staining revealed that IDV-specific antigens were present in the alveolar epithelium and bronchiolar epithelium in the direct inoculated and also in positive sentinel animals [115]. Murine (DBA/2 mice) model of IDV demonstrated productive viral replication in the extra-pulmonary organs such as intestines, liver, and kidney in addition to the upper and lower respiratory tract with peak replication at 4 dpi with no obvious clinical signs and weight loss [116].

**Figure 3:**
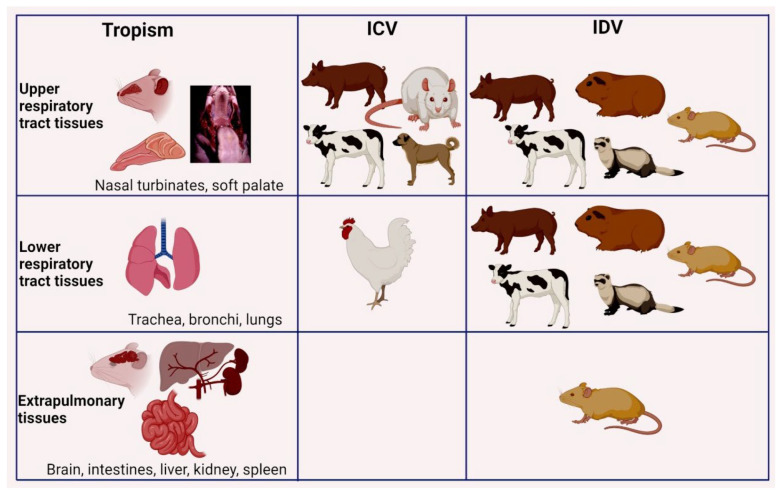
A synopsis of the tissue tropism demonstrated by the seven-segmented viruses, ICV, and IDV in different experimental animals based on the literature review. The illustration was created using Biorender.com, accessed 30 October 2021.

The possibility of the wild animals as a potential host for IDV has been tested by conducting an infection study in feral swine which demonstrated virus shedding at 3–5 dpi, seroconversion at 21 dpi, and also transmitted the virus to fifty percent of the in-contact animals. Immunohistochemistry also revealed the viral antigen in the respiratory epithelium of the trachea, soft palate, and lungs [97]. A summary of the tissue tropism of ICV and IDV in different experimental animals was shown in Figure 3, which shows that experimental animals such as ferrets, guinea pigs, and mice were used for IDV studies compared with ICV.

## 4. Clinical Manifestations/Pathobiology of ICV and IDV in Principal Host Species

As already discussed, ICV can cause clinical outcomes from mild to moderate respiratory infection in adults to severe pneumonia in children. The symptoms of ICV infection detected in 38/892 military recruits in Eastern Finland included cough and rhinitis without any fever [117]. A clinical study of a small outbreak of ICV during November–December 1978, among employees, demonstrated that ICV illness involved muscle aches, fever, headaches which is clinically indistinguishable from IAV, and ICV patients took longer to recover compared with employees with IAV infections [105]. However, clinical manifestations of ICV in children are reported to have more variable outcomes from upper and lower respiratory infections (cough, rhinitis, wheeziness without temperature, respiratory distress) to encephalopathy [56,118]. A follow-up study of ICV in children in March 1981, showed that four secondary and two tertiary infections had occurred in two years of survey time. The children exposed to the infections exhibited mild respiratory signs, fever, nasal discharge, etc. The incubation period was ≤5 days and virus shedding lasted for 22 days [119]. These facts indicate the inadequacy of the pre-existing immunity and also the in vivo stability and long-term shedding of the ICV. Community-associated pneumonia linked to ICV has been increasingly reported in children 1 year to 14 years in Spain and Italy, characterized by fever, cough, rhinitis, low oxygen saturation, low WBC count, C-reactive protein and procalcitonin, and alveolar interstitial pneumonia [51] with reports of co-infections with adenovirus, RSV-B, and influenza A and B infections [120]. A six-month hospital study in Japan recorded a similar pneumonia rate and wheezing for influenza virus and human metapneumovirus [121]. Despite producing a good antibody titer after each infection, the occurrence of ICV reinfection indicates the genetic variability of the ICV strains, causing antigenically diverse ICV strains to co-circulate over time [119].

In the case of IDV, clinical case presentations were not yet reported. A pathogenesis study conducted by intranasal inoculation of D/bovine/C00046N/Mississippi/2014 in IDV seronegative calves demonstrated viral shedding in nasal swabs from 1 to 6 days post-infection and seroconversion at 6 dpi with high titer (640). Clinical manifestations include dry cough, mucoid nasal discharge, serous ocular discharge, lethargy, adventitious lung auscultation sounds, etc., compared with the uninfected animals. Additionally, the complete blood count demonstrated neutrophilia characterized by a significant increase in the segmented neutrophil population, whereas other parameters were not different between the two groups. The clinical signs were very mild, and there was no difference in the heart rate, respiratory rate, and rectal temperature in the infected versus mock-infected animals [122]. This study also demonstrated a gradual decrease in the virus from upper to lower respiratory tract with a high titer of IDV in the upper respiratory tract (nasal ethmoid turbinates) compared with lower respiratory tract tissues (right medial lung) by q-PCR [122]. Additionally, IDV could transmit from directly inoculated animals to sentinel animals by contact, and the contacted animals seroconverted as early as 9 days post-exposure [122]. 

The histopathology of the IDV-infected cattle demonstrated lesions mostly confined to the upper respiratory tract such as suppurative rhinitis characterized by the mucopurulent exudate over the respiratory epithelium, neutrophil accumulation in the submucosa, neutrophil exocytosis, and multifocal loss of the ciliated epithelium. Similar lesions were observed in the tracheal epithelium with mild neutrophilic infiltration, neutrophil exocytosis, and mild lymphoplasmacytic tracheitis [114]. No lesions were observed in the tracheobronchial lymph node [114]. Intracytoplasmic localization of IDV-specific antigen by IHC was majorly observed in the respiratory epithelium of nasal turbinates and trachea with weak positive staining in the respiratory epithelium of the airways in the lungs [114].

## 5. Bottlenecks for Viral Adaptation and Interspecies Transmission

Though cattle and swine are the most susceptible hosts of IDV, species specificity has an important role in sustained interspecies transmission. An experiment to test the species specificity of the IDV used a co-mingling pig-calf experiment using D/swine/Oklahoma/1334/2011 and D/bovine/Texas/72/2017 viruses. The sequence identity of HEF of these two viruses at the nucleotide and amino acid levels are 97.64% and 98.04%, respectively. Both the viruses replicated in the direct inoculated animals irrespective of species of virus origin. However, interspecies transmission depended on the species of virus origin, in that D/swine/Oklahoma/1334/2011 transmission from calf to pig occurred in only 1/3 pigs, whereas pig to calf transmission of D/bovine/Texas/72/2017 occurred in 3/3 calves with the appreciable virus titers by virus isolation and seroconversion. On the other hand, pig to calf and calf to pig transmissions of D/swine/Oklahoma/1334/2011 and D/bovine/Texas/72/2017 viruses neither yielded any replicable virus nor seroconverted [123]. Ducatez et al. also determined that current species specificity is the most important driver and is considered to have the highest weightage among the seven other driving factors that led to the IDV emergence in Europe [124]. Though inconclusive, receptor preferences of different IDV lineages could be a limiting factor in different species, and more studies are needed to understand how slight phenotypic changes can contribute differently to cross-species transmission.

## 6. Phylodynamics of Seven-Segmented Viruses

Influenza C and D genomes comprise seven RNA segments coding for polymerase proteins PB2, PB1, and P3, hemagglutinin esterase fusion protein (HEF), nucleoprotein (NP), matrix (M), and a non-structural protein (NS). The segments PB2, PB1, P3 are long and form heterotrimeric polymerase complex and are similar to IAV and IBV. Compared with IAV and IBV, ICV/IDV polymerases were less studied. While the active polymerization site is present in PB1, gene segments PB2, and P3 harbor cap-binding and endonuclease domains, respectively, for transcription initiation via cap-snatching. The NP protein functions with polymerase complex and RNA to form viral ribonucleoprotein complex (vRNPs). The structural protein P42/M segment undergoes alternative splicing to code for two proteins responsible for ion protein channel activity (M2) and morphology (M1). NS protein also encodes two proteins NS1 and NS2 responsible for interferon antagonism and nuclear export of RNPs. Segment 4 encoding HEF glycoprotein is responsible for receptor binding, fusion, and receptor destroying activities and is the most studied protein. The structural and functional details of the ICV and IDV genomes are described in detail under Section 10.

Early evolution studies have shown that the HEF protein of ICV shares a more distant relationship with both A and B influenza viruses, comparable to that between IAV and IBV [125,126]. Based on the HEF sequences, there are six distinct lineages represented as C/Taylor, C/Aichi, C/Mississippi, C/Sao Paulo, C/Kanagawa, and C/Yamagata [127]. A surveillance study involving 3300 Scottish respiratory samples revealed less ICV than IAV and IBV, and also found multiple reassortant lineage viruses [73]. A comparison of sixteen ICV genomes using SDS-PAGE and OGN fingerprinting in Japan demonstrated two major groups and suggested that multiple distantly related ICVs co-circulate at the same time [128]. Japanese ICV strains (C/Kyoto/41/82, C/Nara/82, C/Hyogo/1/83) isolated between February 1982 and December 1983 were distantly related to the old Japanese strains; however, they shared a 98.4–98.5% sequence identity with the US strain (C/Mississippi/1/80), showing the intercontinental transmission [129].

Recent studies in different parts of the world have demonstrated that Sao Paulo and Kanagawa lineage ICV circulated in Germany around 2012–2014 [130]. In South Korea, the first report on the ICV incidence was reported in young children sampled between 2013–2016 and found that C/Sao Paulo/387/82-like viruses circulated in South Korea and C/Seoul/APD462/2015 were phylogenetically related to C/Victoria/2/2012 and C/Tokyo/4/2014 strains [59]. A surveillance network study for acute respiratory infections in Minnesota, the US from May 2013–December 2016 revealed 19–22 respiratory pathogens and found the seasonal occurrence of ICV with year-to-year variability. HEF belonging to both C/Kanagawa and C/Sao Paulo was sequenced in 37/70 samples, which include both inpatient and outpatient cases [131]. Similarly, ICV strains from India isolated between 2009–2015, showed close phylogeny of HEF with Sao Paulo and Kanagawa lineages whereas PB2, PB1, M, and NS clustered with C/Yamagata and P3, and NP clustered with C/Mississippi lineages, respectively [132]. Another study from Singapore indicated that ICV strains isolated in 2006 belonged to the C/Kanagawa lineage [133]. Australia also witnessed high rates of ICV infection between 2012 and 2014, and most of these strains belonged to Sao Paulo and Kanagawa lineages [134,135].

**Figure 4:**
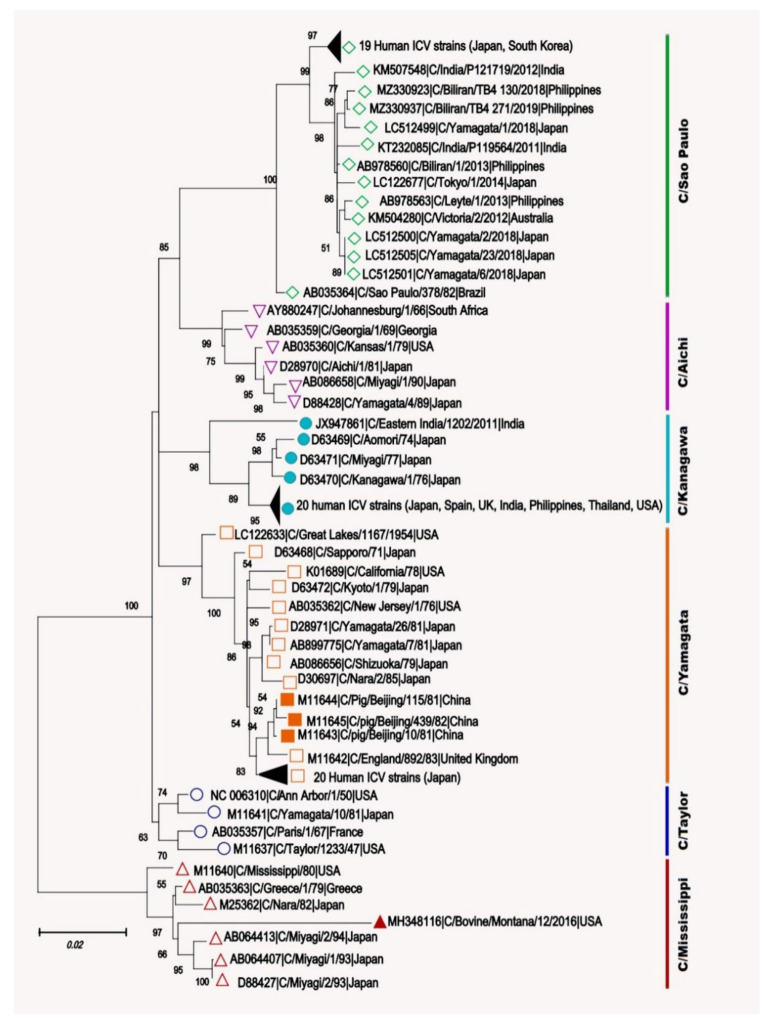
Phylogenetic tree of the complete protein-coding nucleotide sequences of the ICV-HEF. The evolutionary history of ICV-HEF was inferred using Maximum-likelihood analysis in MEGA X [136]. Tamura 3 parameter model + Gamma Distributed (G) [137] model was used with a bootstrapping of 1000 replicates for the analysis. The analyses involved 94 sequences of ICV strains of human, bovine, and swine origin. Different lineages of ICV are color-coded and marked as follows. Green open diamond—C/Sao Paulo; purple open inverted triangle—C/Aichi; Cyan filled circle—C/Kanagawa; Orange open square—C/Yamagata; Blue open circle—C/Taylor; Red open triangle—C/Mississippi. The strains of bovine ICV (Red filled triangle) and swine ICVs (Orange filled square) belonged to C/Mississippi and C/Yamagata, respectively. A scale representing evolutionary distance that indicates the number of nucleotide substitutions per site is also shown. Bootstrap values are shown above branches to the left of major nodes.

The bovine strain C/bovine/Montana/12/2016 isolated from a sick calf with the respiratory disease showed a close phylogenetic relationship with human ICV strain C/Mississippi/80 with a sequence identity of 97.1% [95]. Overall, bovine ICV isolates shared a high nucleotide sequence identity of 98% with each other and 95% to human ICV strains [22]. A phylogenetic analysis of the complete protein-coding nucleotide sequences of the HEF segment of ICV was performed conducted using MEGA X, and results are shown in Figure 4. The swine ICV genomes clustered with C/Yamagata human ICV strains, whereas the bovine ICV clustered with C/Mississippi lineage viruses.

Phylogenetic analyses of the D/bovine/Oklahoma/660/2013 (bovine IDV) with the representative influenza A, B, and C viruses, Thogoto virus, and infectious salmon anemia virus demonstrated that all the seven segments of bovine IDV clustered together with the human ICV, suggesting that IDV diverged from human ICV, after the major divergence from IAV and IBV [138]. Phylogenetic analyses also showed that bovine and swine influenza D viruses did not undergo reassortment with human ICVs, even though reassortment is quite frequent in human ICV. The divergence time of the five genes estimated by the phylogenetic analysis demonstrated that the estimated mean time to the most recent common ancestor (t-MRCA) of IDV and ICV was 334, 1299, 541, 545, and 610 years for PB1, HEF, PB2, P3, and NP, respectively [86]. 

**Figure 5:**
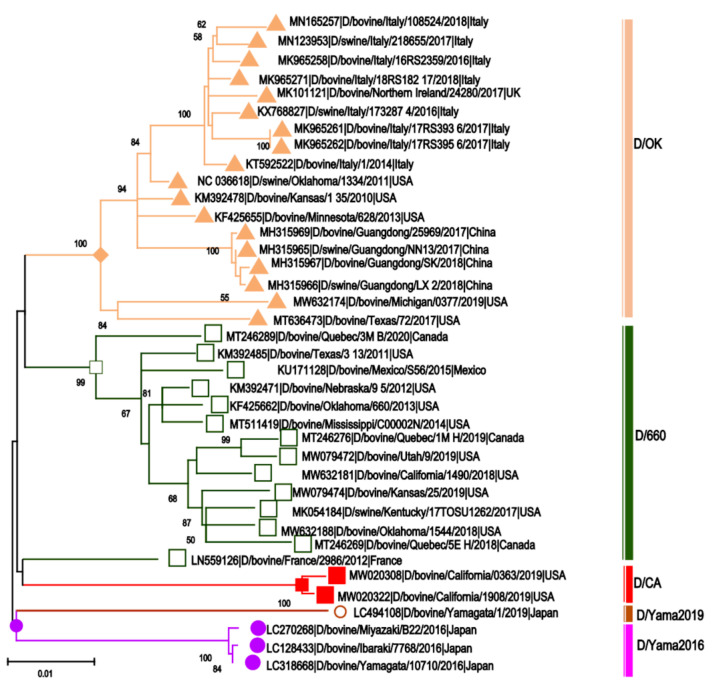
Phylogenetic tree of the complete protein-coding nucleotide sequences of the IDV-HEF. Phylogeny was inferred using maximum-likelihood analysis in MEGA X [136], with a bootstrapping of 1000 replicates. The tree was derived using Hasegawa-Kishino-Yano model + Gamma Distributed (G) [HKY + G] model. The analyses involved 38 sequences of IDV strains of bovine and swine origin from different geographic locations. Different lineages of IDV are color-coded and marked as follows: D/OK (Light Orange filled triangle), D/660 (green open square), D/CA2019 (Red filled square), D/Yama2019 (brown open circle), and D/Yama2016 (Purple filled circle). A scale representing evolutionary distance that indicates the number of nucleotide substitutions per site is also shown. Bootstrap values are shown above branches to the left of major nodes.

According to the global distribution of IDV, five influenza D lineages have been characterized based on their distinct genetic and antigenic properties, represented by the D/OK (D/swine/Oklahoma/1334/2011, D/660 (D/bovine/Oklahoma/660/2013), Japanese strains-D/Yama2016 (D/bovine/Ibaraki/7768/2016) [137], D/Yama2019 (D/bovine/Yamagata/1/2019) [76], and the recently identified D/CA2019 (D/bovine/California/0363/2019) [139]. D/660 and D/OK lineages were the most common lineages in North America. The phylogeny of different IDV strains were shown in Figure 5. 

## 7. Reassortment and Species Specificity

The genomic analyses of the ICV isolates of swine origin were compared with the human ICV isolates from 1947–1981 by oligonucleotide mapping and RNA migration patterns, and found that the swine genomes were distantly related to the human ICV strains. Though closely related to each other, the swine ICV genomes, C/pig/Beijing/10/81 and C/pig/Beijing/32/81 isolated in the same day/year/place differed by several mutations in the RNA segments 1 and 2, suggesting the possibility of genetic reassortment. The studies on the genetic relatedness of the ICV strains isolated in different parts of the world over 32 years suggest that ICV is much more stable with fewer variations compared with IAV/IBV [140]). It is worthwhile to note that human ICV strains were not reported from Beijing, China, till 1981 when swine ICV strains were isolated from the abattoir pigs. The genetic analyses of the swine ICV sequences from 1981 and 1982, also demonstrated mutations in the RNA segments 1, and 2. However, a major drift scattered over the whole genome was found when strains C/Pig/Beijing/32/81 and C/NJ/76 were compared [37]. The close homology of the swine and human ICVs and their serological and genetic similarities suggest the possibility of pigs as a natural host for ICV [37]. 

**Figure 6:**
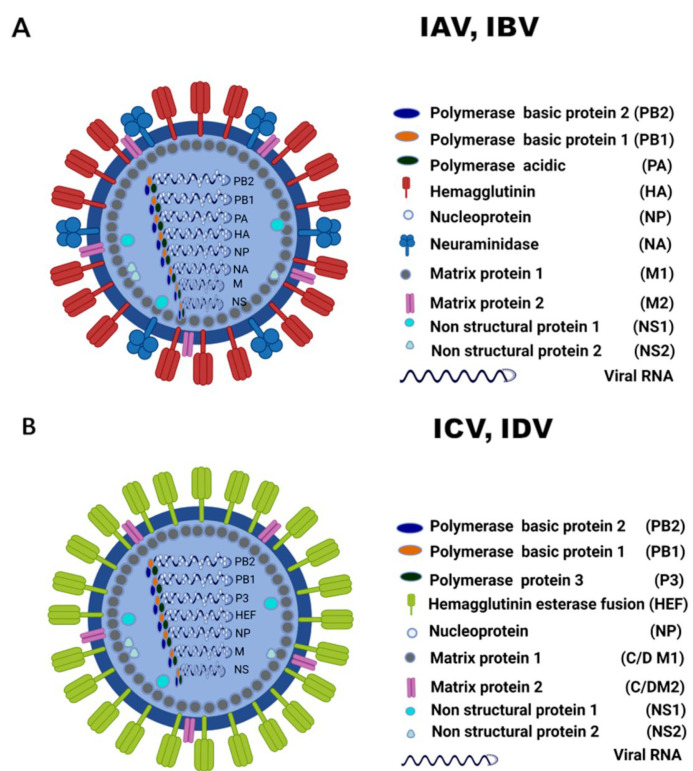
The structural differences of eight segmented influenza viruses and seven segmented influenza viruses. The different proteins are marked with a description on the right. (**A**) Eight-segmented influenza viruses (IAV, and IBV) with separately encoded Hemagglutinin (HA) and neuraminidase (NA) proteins for receptor binding and destroying functions, respectively. (**B**) Seven-segmented influenza viruses with Hemagglutinin–esterase fusion protein that combines the dual function of HA and NA. The graphical illustration was created using Biorender.com, accessed 30 October 2021.

The similarity index to determine whether the host species can influence ICV-HEF’s codon usage pattern indicated that swine induced higher selection pressure than bovines and humans, as demonstrated by the similarity indices [141]. Structural studies of the selection pressure of the antigenic sites of the different ICV lineages showed that the similarity indices of the C/Kanagawa lineage were the highest and the Sao Paulo lineage showed the lowest [141]. A phylodynamics study involving 106 complete genomes over the 68 years since the ICV discovery indicated that no virus has been isolated that belonged to C/Taylor after 1967, Aichi lineage viruses isolated before 1990 were not detected after 1992, and the Mississippi lineage viruses were also isolated before 1990 (Figure 6A). The Kanagawa and Sao Paulo lineage viruses were currently seen co-circulating across the world [142]. The porcine strains belonged to C/Yamagata, whereas the bovine ICV belonged to the C/Mississippi lineage which became extinct in humans [143]. It is interesting how bovine ICV/2016 demonstrated the characteristics of Mississippi lineage after an extinction period of 25 years. Several antigenic and phylogenetic studies have shown that reassortment of two influenza C viruses occurs frequently and the genome arrangement of these reassortant ICVs influences their transmission within and between species [144].

### 7.1. Reassortment between IDV Lineages

Natural reassortment between D/OK and D/660 lineages/clades has been noticed during the phylogenetic analyses of the four respiratory samples collected from the Quebec cattle of Canada in 2018–2020 [145]. Furthermore, a recent study also demonstrated that PB2, PB1, P3, NP, and NS segments of IDV are under stronger selection pressure in bovine species than swine, and hence frequent reassortment can occur. On the other hand, the HEF and P42 segments of swine IDV exhibited more non-synonymous substitutions [146]. A combined molecular and serosurveillance study in a single-order buyer facility in Mississippi indicated high seroprevalence, along with 32 new IDV isolates derived from healthy and sick animals, of which 5 were reassortants, showing that active genetic reassortment has occurred in IDV [147].

### 7.2. Reassortment between ICV and IDV

The in vitro reassortment experiments involving the human ICV or the animal IDVs always generated homologous reassortant viruses either between the human ICVs or the animal IDVs, but clearly not heterologous reassortant between the human ICV and animal IDV, showing that IDV is different from ICV, which is one of the reasons behind the classification of IDV as a separate genus in the *Orthomyxoviridae* family [138]. An in vitro reassortment experiment was conducted using two human ICVs, C/Taylor/1947 and C/Johannesburg/66 (C/JHB) with D/bovine/Oklahoma/660/2013 (bovine IDV) and D/swine/Oklahoma/1334/2011 (swine IDV) to test whether these distantly related ICV and IDV viruses can produce antigenic variants by reassortment. This co-infection study, with pairwise virus combinations of the human ICV and IDV, generated reassortant viruses with segments derived from swine or bovine IDV [138]. Another in vitro reassortment co-infection study using C/Johannesburg/66 (C/JHB) and D/swine/Oklahoma/1334/2011 (D/OK) at different combinations of MOI also yielded the viruses with segments of D/OK origin, despite the higher infectivity of ICV over IDV at 24, 48, and 72 h post-infection [138]. 

The 3′ and 5′ non-coding (NC) regions of sequences of IDV genome segments were similar to the human ICV genome with one nucleotide exception (position 5 from the 3′-terminus) and polymorphism at position 1 of the 3′terminus. Hause et al. showed that HEF, M, and NS segments of the IDV have U at the 3′-terminal position, whereas PB2, PB1, P3, and NP segments possess (C) [82]. However, Ishida et al. demonstrated no polymorphism in this site [148]. This nearly identical NC region at both ends of ICV and IDV segments opens the possibility of viral segment reassortment [148,149]. Recently, it was demonstrated that approximately 70 and 73% of influenza C and D viruses also package eight ribonucleoproteins (RNPs) segments similar to influenza A and B viruses, which needs further investigation [150]. 

## 8. Broad Cell and Tissue Tropism of IDV over ICV

Earlier attempts to isolate the egg-adapted ICV in the human embryonic lung (HEL), embryonic kidney cultures from human, or bovine origin were not successful. The failure of influenza C replication in cell culture systems permissible to IAV and IBV has been discussed by Green et al., which shows the differential pathogenesis of this human pathogen compared with the other eight-segmented influenza A and B viruses [151]. Monkey kidney cells were used for ICV propagation [152,153]; however, the majority of the ICV strains failed to adapt to mammalian cell cultures [154]. Primary chick embryo kidney (PCEK) cell cultures were used to grow ICV to high titers in the presence of 2 µg/mL [155]. Canine kidney cells have been first used for the propagation of influenza C by Nerome, K and Ishida, M [156], and these researchers also established monkey kidney cell line (LLCMK2) and found out that these cells can yield high ICV titers compared with chicken embryo amniotic inoculation [157]. ICV strain C/JJ/50 showed developmental abnormalities in feathering and found that day 10 embryos yielded high titers than day 12 [158]. The human melanoma cell line (HMV-II) possessed 2–4 times more receptors than MDCK and hence were used to propagate ICV strains in the presence of 5–20 µg/mL of Trypsin [159]. However, it was found that HMV-II grown viruses (C/Yamagata/4/88, C/Yamagata/7/88) were antigenically different from the egg grown viruses [160]. The increased permissibility of MDCK II cells compared with MDCK I cells for ICV is due to the amino acid change from Threonine to Isoleucine at position 270 of the HEF protein [161].

Compared with ICV, IDV has broad cell tropism owing to its increased binding avidity towards diverse glycans and can grow not only in continuous cell lines, but also in most of the primary cells from bovine [162], caprine [163], equine, and porcine species [164]. A comparison of in vitro cellular tropism of the IDV to ICV, determined by RT-PCR in cell lines of different species origins, such as swine testicle (ST) cells [165], (MDCK), green African monkey kidney cells (Marc-145), human rectal tumor (HRT-18G), baby hamster kidney (BHK-21), and porcine kidney (PK-15) cells, showed that IDV replicated to appreciable titers in all, with minimal replication in BHK-21 and PK-15 cells [82]. In ST cells, IDV caused influenza-like cytopathic effects by 3 days post-infection [82]. It should be also noted that IDV can grow both at 37 and 33 °C, showing its tropism to both lower and upper respiratory tracts compared with ICV, which is more suited to grow at lower temperatures [82].

The structural basis for the difference in the broad cell tropism exhibited by the IDV is due to the receptor preference, affinity, and specificity compared with ICV. ICV utilizes sialylated glycan which has 9-*O*-acetylated sialic acids, especially the 9-*O*-acetyl-*N*-acetyl neuraminic acid (Neu 5,9Ac2). IDV and ICV possess sialate *O*-acetylesterase to cleave the 9-*O*-acetyl group. The enzymatic catalytic triad of the C/Johannesburg (C/JHB) and D/swine/Oklahoma/1334/2011 (D/OK) are identical: S71/H369/D365 (C/JHB) and S73/H375/D372 (D/OK) [82]. Other key conserved residues that are critical for the optimal enzymatic function, such as G99/N131; R72/R332 and G101/N133; R74/R342 for C/JHB and D/OK, respectively. These conserved enzymatic residues suggest that IDV and ICV utilize 9-*O*-acetyl sialic acid as the cellular receptor for infection [82]. The common amino acid residues responsible for binding to the 9-*O*-acetyl groups in the receptor-binding pocket of ICV (C/JHB) are Y141, F239, Y241, R250, and R302, whereas IDV utilizes all these residues except for Y141 and R250, which were replaced by tyrosine residues at 143 and 256 positions for IDV [82]. Additionally, the amino acid residues at the 5-*N*-acetyl binding pocket of ICV is L198, whereas in IDV it is W201, which causes the binding pocket smaller due to the large aromatic sidechain. These changes may alter the binding preferences of IDV, which could be accountable for the difference in the cell tropism between ICV and IDV [82].

Ovine explants from nasal and tracheal tissue and the lungs supported IDV infection, as evidenced by the productive replication in these tissues with swine strain of IDV replicating 1–2 logs less than the bovine strain of IDV. Further a virus binding assay on histological tissue sections from nasal tissue, submucosal glands, pharynx, trachea, and lungs demonstrated a high affinity to nasal tissue, glands, and pharynx in bovines; glands, and pharynx in the case of small ruminants; and glands only in horses. It is interesting to note that swine tissues did not show any binding in any of these tissues [166]. However, a tissue microarray involving nasal tissue, pharynx, upper and lower trachea, and lung tissues with recombinant HEF protein (HEF S57A) with deficient esterase activity showed high binding to the epithelium of nasal, pharyngeal, upper and lower trachea in cattle, and only to the pharyngeal and nasal epithelium in pigs. In the case of sheep, and goats, the protein histochemical staining was comparable with almost the same level of binding to the nasal and pharyngeal epithelium, whereas the binding was mostly localized in the submucosal glands of nasal and pharyngeal tissue in horses [167].

## 9. Origin, Classification, and Morphology

A comparative study of the RNA genomes of influenza A, B, and C viruses found that ICV also possesses a segmented single-stranded RNA genome similar to IAV and IBV and its RNA base composition (>30% Uracil) is almost comparable to A and B viruses [168]. A study by Kox and Kendal demonstrated that ICV is a segmented single-stranded RNA genome [169]. The structural differences between the eight (IAV, and IBV) and seven (ICV and IDV) segmented influenza viruses are shown in Figure 6A,B. In 1950, Hirst G. K compared the receptor specificity and replication pattern of the prototypic 1233 ICV strain to similar hemagglutinating myxoviruses (MUMPs, NDV, influenza) and found that ICV possesses a distinct receptor destroying enzyme [170]. Later on, experiments with fetuin and sialyl lactose as substrate confirmed the absence of neuraminidase and also found that heat stability of the hemagglutinin of ICV is more pronounced compared with IAV [171].

The morphology of A, B, and C influenza viruses were studied using electron microscopy and it was found that most of the IAV particles were roughly circular with projections of sizes 1000–1500 Å, and few were filamentous. The IBV particles were similar to IAV, except for some with a kidney/ring shape, whereas ICV demonstrated spherical to filamentous forms, with hexagonal structures on the surface [172,173]. Further studies have shown that clinical isolates of IAV were usually filamentous. IAV, being highly pleomorphic, tends to display differential morphology ranging from filamentous to spherical, with frequent ovoid or bacilliform intermediates which are dependent on the M1 expression [174]. The RNAs of influenza C strains were studied using radiolabeled strains of C/JHB/1/66, C/JHB/1/67, and C/Yamagata/1/64 and found that their RNA migration patterns were comparable to the already well-characterized A/WSN virus [175]. Further studies of the three structural polypeptides (gp 88/HEF, M, and NP proteins) of five isolates from 1964–1981 originated from USA and Japan showed similar migration rates, but different from the prototypic strains C/Taylor/1233/47 and C/JJ/50. Furthermore, in 1983, the other five polypeptides (C1-5) encoding viral proteins were studied using the C/JJ/1950 strain propagated on MDCK cells [176]. Similarly, MDCK cells infected with human and swine ICV strains were also used for studying the virus-specific polypeptides and found that viral proteins of the human and pig ICV strains between 1947–1981 are highly conserved [177]; however, it was shown that ICV is highly susceptible to self-disintegration and is fragile when compared with A and B viruses [178]. Cloned cDNA of segment 4 of the C/Califoria/1978 was prepared and found that ICV hemagglutinin shares structural and functional similarities to the HA of IAV and IBV, suggestive of a common precursor [125]. 

The fatty acid linkage of ICV-HEF is compared with the HA of IBV and found that ICV-HEF is acylated with stearic acid whereas IBV is acylated with palmitic acid [179]. The occurrence of more hydrophobic stearic acid as protein-linked fatty acid explains the hexagonal arrays on the ICV membrane [179]. The electron cryotomography of vitrified virions demonstrated that ICV particles are pleomorphic from spherical to filamentous nature with variable sizes. Additionally, the virions are covered by HEF glycoprotein in open hexagonal lattices. The presence of clustered HEF glycoprotein layer alone is sufficient for the virus budding; however, these spherical particles may or may not have the dense matrix layer with dense RNP assemblies inside [180]. The size of the particles with the matrix layer looks slightly smaller size as the virion morphology and size depend on the packaged RNP assembly to the matrix layer [180]. The receptor-binding site is closer to the apex. Even though both the receptor-binding site and the esterase active sites open within the lattice, the antigenic sites between the trimer interface could destabilize the lattice contacts [180]. The folding and binding of ICV-M1 to polymerize to form positively charged ring-like or filamentous structures and its electrostatic interactions with negatively charged plasma membrane components and tubules to form tubulation/invaginations determine the diameter of the viruses and hence helps in viral budding and stabilizing virions [181]. Both N and C termini of ICV-M1 are required for interaction and tubulation, respectively [181]. 

The motility of ICV was studied using surface reflection interference contrast microscopy and found that ICV with filamentous morphology demonstrated a unidirectional movement with frequent turning by flapping compared with the viruses with spherical morphology moving in gliding and short crawling. Although indirect, ICV-M1 has a role in filamentous morphology. Among other different factors, it was found that the receptor destruction by the viral esterases along the trajectory has a key role in the virus motility, and the level of receptor density present at the front and back of the virus steers the direction of motility [182]. Post-translational modifications such as *N*-glycosylation and phosphorylation are required for viral replication, whereas the loss of palmitoylation did not affect the ICV replication [183]. The effect of non-acylated HEF (acylation site removal at the cytoplasmic tail) on viral replication fitness was studied and found reduced fusion activity with slightly reduced viral titers; however, the morphology of the viruses did not change with the mutation [184]. 

In the case of IDV, such morphology and biological studies are limited. The morphological characteristics of D/Yamagata/10710/2016 and C/Ann Arbor/50 using scanning transmission electron microscopic tomography demonstrated that more than 70% of the influenza C and D viruses also follow the RNP packaging in a 1 + 7 pattern, similar to IAV and IBV [150]. Furthermore, IDV (D/bovine/Yamagata/10710/2016) also demonstrated pleomorphic morphology ranging from spherical to elliptical or filamentous forms, with a hexagonal arrangement of HEF glycoprotein on the envelope similar to ICV [150]. However, cord-like structures found on the ICV-infected cells were occasionally present in the case of IDV, suggesting the type-specific genetic differences in the M segment [150].

## 10. Genome Structure

### 10.1. Influenza C Virus

Segments 1,2,3-Polymerases: The ICV genome codes for seven RNA segments of negative polarity. According to the RNA migration patterns, it was earlier deduced that the length of RNA segments 1, 2, 3, 4, 5, 6, 7 are 2400, 2400, 2200, 2100, 1850, 1200, and 960 nucleotides, respectively [185]. Earlier, SDS-PAGE of ICV-infected MDCK cells generated a series of viral polypeptides that include PB2, PB1, P3, HEF, NP, NS, and M [176,177]. The segments PB2, PB1, P3 are long and form a heterotrimeric polymerase complex. The polymerase segments PB2, PB1, and P3 of C/JJ/50 were cloned, sequenced, and compared with IAV and IBV polymerase proteins. It was found that one long ORF is present, similar to IAV and IBV. PB2, which is considered as a determinant of host range for IAV is not much studied in the case of ICV. However, a mutation of T28C changes the aa Leu to Phe, and has been found to have associated with persistent phenotype in vitro [186]. Segment 1 encoding PB2 of ICV shared a 25% sequence identity with PB2 of A and B viruses, PB1 shared a 40% sequence identity and hence is the most conserved among the A, B, and C viruses. Compared with IAV and IBV, the RNA-dependent RNA polymerases of ICV were less studied. Hengrung et al. reported the crystal structure of ICV polymerases and reported a closed conformation, indicating a transcription preactivation state [187]. The segment 3 protein shares around 25% sequence identity; however, the acidic charge at neutral pH is absent, unlike the polymerase acidic protein of the A and B viruses, and hence this polymerase is designated as P3 protein for ICV [188]. 

Segment 4, HEF: Segment 4 encoding HEF glycoprotein (designated earlier as gp88) of ICV possesses 2073 nucleotides in length with 655 amino acid polypeptides, with a molecular weight of 72,063 Da, excluding the eight, predicted glycosylation sites. The association of receptor destroying enzyme (RDE) with HEF glycoprotein was indicated by the decrease in the RDE activity when ICV was treated with various doses of Trypsin [189]. Even though this protein lacks sequence identity for the major part, it structurally shares features with HA protein of IAV and IBV, with three stretches of hydrophobic amino acids indicative of signal peptide, fusion protein, and membrane anchorage properties [125,126]. Further studies with recombinant protein indicated that segment 4 encodes for viral glycoprotein, which has receptor-binding and receptor-destroying activities [190]. Bromelain digestion of MDCK cell grown C/JHB/1/66 produced a soluble form of the spike protein which possessed both receptor binding and receptor destroying properties and also revealed a trimeric structure for the spike protein [191]. Herrler et al. purified the glycoprotein by octylglucoside treatment of ICV, followed by sucrose gradient ultracentrifugation and found that HEFhas hemagglutination, receptor destroying, and fusion activities and proposed to designate this glycoprotein as HEF for hemagglutinin (H), esterase (E), and a fusion factor (F) [192]. HEF possesses a short N terminal signal peptide of 14 aa, an ectodomain of 612 aa, a transmembrane region of 26 aa, and a very short cytoplasmic tail of 3 aa. HEF protein cleaves to form HEF1, which is the N terminal 432 aa, and HEF2 which constitutes the hydrophobic fusion peptide, a transmembrane domain, and a cytoplasmic tail. 

Compared with HA, the HEF protein is comparatively invariant between the six lineages. An alignment of the HEF of the six lineages of ICV revealed that all the residues pivotal for the structure, glycosylation sites, hydrophobic fusion peptide of the N terminal of the HEF2, receptor binding, and receptor destroying domains are conserved. There are around 35 variable aa residues located in HEF1 and 7 in the smaller subunit of HEF2. Three important localized small regions of variable amino acids at 61–65 near the esterase domain, and the two regions 165–172, and 190–195 located near the receptor-binding domain are subjected to antigenic drift and are targets for antibody epitopes [161].

*N*-acetyl 9-*O*-acetyl neuraminic acids are the receptor determinants [193], and the viral enzyme responsible for esterase activity is 9-*O*-acetyl esterase, which hydrolyzes acetyl groups from the natural receptor, 9-*O*-acetyl neuraminic acid. Para-nitrophenyl acetate (PNPA) is a suitable substrate in vitro. The ICV esterase is a serine hydrolase, and such enzymes contain a catalytic triad containing serine, histidine, and aspartic acid residue. In the case of ICV, S71, H368/369, D261 could form a catalytic triad [194]. Two other substrates, a-naphthyl acetate and a-naphthyl propionate, were also found to be sensitive to ICV esterase, and a-naphthyl acetate-based assay could detect esterase activity in vitro as early as 8 h post-infection [195]. The temperature sensitivity of the HEF was studied, and it was found that HEF expression on the cells increased at 33 °C compared with 37 °C, due to more efficient oligomerization. Additionally, the HEF membrane fusion and replication is more efficient at 33 °C because of the better biological activity of the viral RNA polymerase and HEF [196].

Segment 5/NP: The nucleoprotein encapsidates the viral genome and is associated with each viral segment by packing together along with the viral polymerases to form the viral ribonucleoprotein complexes (vRNPs). The nucleoprotein (NP) protein of IAV and IBV have long flexible N terminal regions and ICV differs from both of these by its shorter N terminal region [197]. However, ICV possesses an extended C terminal region which is 54 and 51 aminoacids longer than IAV and IBV, respectively, with a long bipartite nuclear localization signal (NLS) involving residues R513-K549. A particular motif, KKMF in the cytoplasmic tail domain (CTD) of this region is responsible for the polymerase activity and also for binding with different importin isoforms for nuclear import as indicated by the impaired RNP function after deleting or mutating the NLS [197]. The NP of IDV and ICV possess 37.3% sequence similarity, and the KKMF motif is not present in the CTD of the IDV-NP; instead, this contains two stretches of 510-RTGAKRR-516 and 531-KKRGR-535, similar to the bipartite NLS of the ICV [197,198] NLS.

Segment 6/P42: Segment 6 codes for an unspliced mRNA, that forms P42protein which is composed of 374 amino acids,. P42 becomes cleaved by signal peptidase to form M1 and CM2, containing N terminal 259 aa and C terminal 115 aa, respectively [199]. M1 phosphorylation is conserved among A, B, and C viruses [200]. Studies with electron microscopy and mutant recombinant C/Ann Arbor/1/50 virus with A24T mutation of the M1 protein exhibited spherical morphology instead of the wild-type filamentous morphology, indicating that M1 is a major determinant of morphology [201,202]. Unlike IBV which codes for unspliced M protein, ICV matrix protein is coded by a spliced mRNA from segment 6. A splicing event codes for 242 amino acid M protein, although the open reading frame can code for 374 amino acid protein [203]. The cytoplasmic tail domain (CTD) of ICV-M2 was studied using the mutants, and it was found that length of the ICV-CM2 cytoplasmic tail or certain amino acids are required for the conformation, stability, and intracellular transport of CM2 [204]. Studies show that ICV-M2 ion channel activity is similar to IAV-M2; type-specific extracellular and cytoplasmic domains are crucial for infectious virus production, as indicated by the chimeric IAV containing the transmembrane domain of ICV-M2 [205]. Though glycosylation-deficient ICV-M2 affects the ion channel activity and affects uncoating and genome packaging [206,207], palmitoylation of ICV-M2 does not affect the replication or transport/maturation of the glycoproteins such as HEF, NP, M1, M2, etc. [208]. 

Segment 7/NS: Nakada et al. reported the complete nucleotide sequence of the smallest segment 7, encoding the NS protein of C/California/78 [209]. The evidence for NS-spliced mRNA to NS1 and NS2 and its differential arrangement to the NS genes of IAV and IBV was also studied [210]. The NS gene responsible for the antiviral responses becomes alternatively spliced to NS1 and NS2/NEP and shares the same function for all the different influenza types. However, the amino acid sequence identity of NS protein between the four types of influenza is low. Nogales et al. compared the recombinant PR8 viruses expressing the heterotypic NS1 protein of A, B, C, and D viruses and found that despite the homologous function, the NS1 proteins of IBV, ICV, and IDV lacked function compared with IAV-NS1 and caused impaired viral replication in A549 cells and attenuated responses in mice [165]. ICV-NS1 splices viral mRNAs, and it is present in the nucleus in the early stages and stays in the cytoplasm during the later stages. Both N and C terminals of NS1 bind to RIG-I; however, the C terminal is responsible for inhibiting RIG-I-mediated signaling and is independent of RNA binding, similar to IAV-NS1 [211]. Nuclear localization of NS2 occurs after synthesis, and then it localizes in the cytoplasm in the later stages. It was also noted that NS2 is present within the viral envelope and the fact that NS2 co-sedimented with vRNPs suggests that it is associated with vRNP and is incorporated into virions [212]. 

### 10.2. Influenza D Virus

The electron microscopic (EM) studies of thin sections of the infected cells demonstrated filamentous budding of the influenza D virions from the plasma membrane. Negative staining EM revealed influenza D as enveloped spherical to pleomorphic virions of approximately 100–120 nm in diameter, with dense projections of 10–13 nm in length and 4–6 nm in diameter on the virion surface. Furthermore, enzymatic analyses detected *O*-acetyl esterase activity using 4-nitrophenol acetate, and also negligible neuraminidase activity analogous to the influenza C virus [82]. 

Whole-genome sequencing of the virions was performed, and a de novo assembly-mapped sequence read to seven contigs of approximately 1000–2400 bp. The open reading frame (ORF) analyses demonstrated a single open reading frame for all the contigs except for the smallest one [82]. Table 3 shows the segment-wise information of ICV and IDV, along with the functions of each genetic segment as shown below.

Compared with IAV, the evolution of IBV and ICV is in a static phase, with a low evolutionary rate, especially at non-synonymous sites [213,214]. These viruses experience strong selection as previous research has shown that most mutations that cause IBV and ICV to diverge from the phenotypically stable state were frequently eliminated [215,216]. The sequence identity of ICV and IDV is less than what was observed among the six different lineages of human ICV. The sequence identities between ICV from different species and against IDV were analyzed using BLASTn and BLASTp and shown in Table 4.

The sequence analysis of the predicted PB1 amino acid sequences, the most conserved influenza virus protein demonstrated that IDV shared 69–72% mean pairwise identity to ICVs and 39–41% to IAV and IBV [82]. However, the PB1 sequences, of IDV were more distant from influenza C viruses [82]. The HEF protein of IDV is a type I membrane containing 664 aa including the signal peptide. The percentage of amino acid sequence identity between HEF, PB2, and P3 of IDV versus ICV is 53%, 53%, and 50%, respectively. Pairwise sequence identity of HEF between IDV and ICV is 53%, compared with 25–30% HA sequence identity between IAV and IBV [82]. On the other hand, segment 3 of IAV and IBV encodes polymerase acidic protein (PA), with a pKa of 5.2. ICV encodes a polymerase with neutral pH (pKa ~7.2), whereas the predicted pKa of IDV is 6.2 [82]. The lowest percent identity observed was for the NS1 protein (29–33%), followed by NP (38–41%) and P42 with 38% identity [82]. Recently, the nucleoprotein of IDV was purified, characterized, and the X-ray structure of the tetrameric D/NP was solved at 2.4 A resolution and found out that the D/NP core is similar to the NP core of the orthomyxoviruses. However, D/NP possesses a carboxy-terminal tail containing bipartite nuclear localization signals (NLS), whereas NLS is located at the amino-terminal tail in IAV and IBV [198]. 

The M expression strategy of IDV is very distinct from ICV. The characterization of the influenza D as a new genus in the Orthomyxoviridae family was based on the M1 protein expression strategy that was completely different from ICV. In the case of ICV, a termination codon is introduced to the M1 open reading frame, whereas the splicing involves the addition of a second exon encoding 4 amino acid peptides, to the primary M1 mRNA (nucleotides 29 to 765 joined to 1050–1062). The variation in the length of the second exon of M1 between ICV and IDV is a novel finding which is the unique difference, and reason to classify as a separate genus [138]. A study conducted in Xenopus laevis oocytes demonstrated that the ion channel activity of DM2 (IDV) is functionally similar to CM2 of ICV [217].

## 11. Viral and Host-Determinants of Influenza C and D Replication

When ICV was discovered, the properties that distinguished it from IBV and IAV were (1) its ability to agglutinate chicken and not guinea pig RBCs, (2) the absence of cross-reactivity to IAV and IBV sera, and (3) complete elution from chicken RBCs at 35 °C for 5 min [218]. Earlier studies have shown that ICV-soluble antigen was detected in the nucleus at 10 h post-infection compared with the 3 h post-infection for IAV and IBV antigens [219]. Protease treatment of ICV at 1–10 μg/mL enhances the infectivity by cleaving the HEF into two subunits [220]. Elastase and Trypsin could cleave the precursor protein HEF into two subunits gp65 and gp30, and increase the virus infectivity; however, chymotrypsin and thermolysin did not possess this cleaving ability [221]. The first evidence for recombination between ICV strains by Racaniello and Palese in 1979 was demonstrated by a mixed infection of MDCK cells with C/JHB/66 and UV inactivated C/JJ/50 [222]. ICV also demonstrated the cell fusion in the presence of proteolytic enzymes at acidic pH with murine erythrocytes at 37 °C, indicating that low pH mediated fusion of ICV envelope with the cell membrane is needed for the virus infectivity [223,224]. The fusion properties of the ICV were studied using the resonance energy assay and found that the fusion pH of most of the ICV strains falls between 5.6 and 6.1 [225]. It was demonstrated that the presence of genomic negative sense strand does not indicate productive virus replication, whereas the plus strand transcription, viral protein synthesis, and associated replication correlate with productive phases [226].

Unlike IAV and IBV, the pH-dependent M1 matrix disassembly of ICV does not need acidic pH, which suggests that uncoating of ICV is similar to paramyxoviruses which use neutral alkaline pH for M1 disintegration [227]. In the case of ICV, a lag in the fusion activity is observed compared with the fast fusion activity of A and B viruses, and the lag is absent at lower pH and comparable to IAV and IBV. The conformation change of the ICV-HEF is the rate-limiting step in the fusion process and it was studied using Trypsin susceptibility and morphological studies and found that membrane fusion occurred at pH 5.7; however, the trypsin induced conformational change occurred late. The rate of the disappearance of the classical hexagonal structure compared with uncleaved precursor glycoprotein (HEF0) was monitored using electron microscopy and found that the disappearance of the structures started at 2 min post pH decline and about 20% of the structures remained after 10 min, which correlates with the fusion activity at low pH, whereas HEF0 did not show any detectable loss of the hexagonal structures [225]. Studies with sialic acid analogs also demonstrated that cleavage of the HEF is not required for the virus–cell fusion [228]. Furthermore, Serrao et al. demonstrated that the lag in the conformational change could be due to the slow formation of a stable ICV-HEF intermediate with structurally flexible C terminal and second chain reversal regions, before folding back to form the six-helix bundle [229]. It was also shown that RDE of ICV is important for effective viral replication as virus-specific RNA and protein were not present after inactivating RDE of ICV, which indicated that the cleavage of the HEF bound receptors is required for the low pH triggered conformational change and fusion [230]. A comparison of receptor binding properties of ICV isolates from Japan, suggested that amino acid residues at 337 (Glu-Lys), 340 (Thr-Tyr), and 347 (Leu-Ser) could be the molecular determinants for the low sensitivity of the hemagglutination to chicken RBCs [231].

In the case of IDV, the hemagglutination titers of swine and bovine strains of IDV were compared against turkey, chicken, bovine, and horse RBCs, and it was found that RBCs (hemagglutination ability in the ascending order) from Turkey > Chicken > Horse showed 85–100% hemagglutinating ability, whereas bovine RBCs showed only 10% hemagglutinating ability [166].

### 11.1. Glycosylation of the HEF as Determinants of Virulence and Transmission

In general, the glycosylation of proteins is key for their stability and function. The importance of ICV-HEF glycosylation in maintaining the antigenic structure and formation of the viral particle was shown using the HEF glycoprotein and its interaction with tunicamycin [232,233]. Tunicamycin did not prevent the virion production; however, at levels >0.25 µg/mL, it impaired the synthesis of HEF by forming a non-glycosylated form of HEF, without affecting NP and M protein synthesis. Though intracellularly stable, these non-glycosylated HEFs are more susceptible to cellular proteases and were unstable outside the cells, degraded, and were not incorporated in the virions. As a result, tunicamycin-treated cells produced virions that lacked surface proteins and lacked infectivity [234]. 

**Figure 7:**
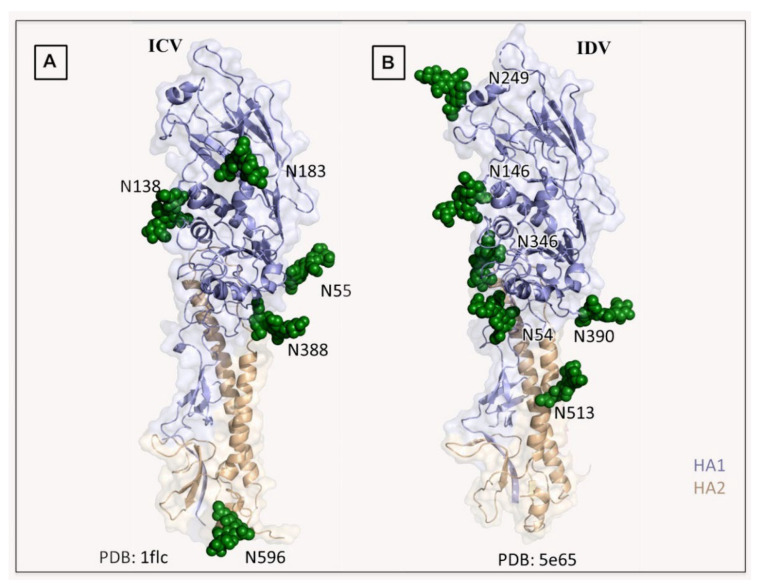
Broad differences of *N*-glycosylation sites of the hemagglutinin esterase fusion protein between influenza C (**A**) and D viruses (**B**). The reference genomes used for the structure modeling are C/Johannesburg/1/66 (**A**) and D/swine/Oklahoma/1334/2011 (**B**). High confident *N*-glycosylation sites (Asn having >40 angstrom^2^ side-chain accessible surface area) were predicted by GLYCAM website. MAN-3 was added to each *N*-glycosylation site (colored green). HA1 and HA2 were colored light blue and wheat, respectively.

Previous studies with glycosylation inhibitors in limiting concentrations suggested that ICV HEF possesses seven glycosylation sites (Asn-X-Ser/Thr) suitable for the attachment of N-linked oligosaccharides, six on HEF1 and 1 on HEF2 [235]. However, our analyses of the ICV-HEF (C/Johannesburg/1/66) for high confident N-glycosylation sites with Asn having >40 angstrom^2^ side-chain accessible surface area suggested only five glycosylation sites as shown in Figure 7. The number of glycosylation sites in HEF protein is seven in bovine IDV (Table 5), six in swine IDV (Table 5), and five in ICV. The predicted glycosylation sites of ICV-HEF from different species such as human, bovine, and pig were found to be similar. The glycosylation sites of ICV and IDV are not similar, except for three out of five sites which are close and shifted by 1–8 amino acids. It is important to note that ICV HEF has two glycosylation sites in the receptor-binding domain, whereas IDV-HEF does not have any glycosylation sites, which may have a role in the virulence and host tropism of this virus [149]. The broad differences of the glycosylation sites of HEF protein of the ICV and IDV as determined by GLYCAM-Web are given in Figure 7. The glycosylation site NKT at N249 is present in some IDV strains, whereas others possess NKA or NRT at position 249. The glycosylation sites of different IDV lineages as predicted by NetNGlyc 1.0 server at threshold 0.5 were shown in Table 5. 

There are various structural differences noticed in the IDV genome compared with the ICV genome: (1) different sialic acid binding sites; (2) sequence variation in fusion domain 1 and 3 of IDV compared with ICV; (3) HEF protein analysis showed similar fusion protein, but the variable transmembrane domain of the IDV-HEF protein; (4) the short 3 amino acid cytoplasmic tail of IDV is CKK and RTK in ICV; (5) the signal peptide of IDV is 16-amino-acid, whereas human ICV possessed 14 amino acids in length; (6) significant sequence variation between the two signal peptides, but the cleavage site is identical; (7) more glycosylation sites compared with ICV. In the case of IDV-NP, the amino acid residue at 381 in the body domain of the NP was found to be a viral determinant for replication fitness in vitro [236].

### 11.2. Receptor Preferences

The experiments with hemagglutination inhibitors, rat α1-macroglobulin (RMG), and bovine submandibular mucin (BSM) indicated that the RDE of ICV is neuraminate *O*-acetylesterase, and the proposed receptor for virus entry is *N*-acetyl-9-*O*-acetylneuraminic acid (Neu5,9Ac2) [237]. Based on this, three common sialic acids such as *N*-acetyl neuraminic acid (NeuAc), *N*-glycolyl neuraminic acid (NeuGc), and *N*-acetyl-9-*O*-acetylneuraminic acid (Neu5,9Ac2) were used for their ability to mediate attachment using human asialoerythrocytes. ICV agglutinated native cells or resialylated cells with Neu5,9Ac2 and not with Neu5Ac and NeuGc whereas IAV and IBV did agglutinate cells resialylated with NeuAc and NeuGc and not with Neu5,9Ac2, thus providing evidence for Neu5,9Ac2 as the receptor determinant of ICV [238]. The treatment of cells containing 9-*O*-Ac-NeuAc with ICV-neuraminate *O*-acetylesterase abolished ICV agglutination and promoted agglutination by IAV and IBV [238]. The ICV-RDE cleaves acetyl groups from C9 of the Neu5,9Ac2. The pre-treatment of LLC-MK2 cells with either neuraminidase or neuraminate 9-*O*-acetylesterase failed ICV infection, whereas the addition of bovine brain gangliosides before infection restored the infectivity, thus confirming that Neu5,9Ac2 is the high-affinity receptor for ICV [239]. A study using lectins showed that ICV RDE can cleave the acetyl group at the ‘N’ position of galactosamine and glucosamine, in addition to the ‘O’ position acetyl groups, and hence these acetylated aminosugars could be a receptor in addition to 9-*O*-acetylated sialic acids [240]. 

The interaction of ICV with human erythrocytes was studied by pretreating the human RBCs with anti-HE monoclonal antibody (complete agglutination inhibition), neuraminidase, or the neuraminate -*O*-acetyl esterase (reduction in virus binding). The proteolytic enzyme treatment with ficin, bromelain, or V-8 protease caused complete inhibition of virus binding which suggested that the ICV receptor could be a glycoprotein. Apart from this, it was also reported that human RBCs harbor fewer amount of ICV receptors compared with rats, chickens, and mice. Further pretreatment of human RBCs with chymotrypsin did not inhibit the virus binding, whereas trypsin reduced the binding by 50% [241]. Rat RBCs have a strong affinity towards ICV, compared with RBCs of other species. The level of affinity for hemadsorption and hemagglutination in the ascending order is rat > fowl > human. The RBCs from day-old chicks, rhesus monkeys, and sheep did not agglutinate ICV [154]. Another study demonstrated that ICV could agglutinate hamsters, chicken, and mouse RBCs, whereas guinea pig, ferret, monkey, and sheep RBCs failed to agglutinate ICV [242]. It was considered that murine erythrocytes possess high levels of Neu5,9-Ac2 than chicken. 

Esterase from ICV was isolated and its substrate specificity was tested against synthetic carbohydrate acetates, esters, and naturally occurring *O*-acetylated sialic acids. Hydrolysis rates noticed in the ascending order are 4-methyl-umbelliferyl acetate > 4-nitrophenyl acetate > alpha-naphthyl acetate > *N*-acetyl 9-*O*-acetyl neuraminic acid. The esterase is classified as sialate 9(4)-*O*-acetylesterase and it hydrolyses (in the decreasing order) *N*-glycolyl-9-*O*-acetylneuraminic acid, *N*-acetyl-4-*O*-acetylneuraminic acid (slower), and 2-Deoxy-2,3-didehydro-*N*-acetyl-9-*O*-acetylneuraminic acid. *N*-acetyl-7-*O*-acetylneuraminic acid, 6-*O*-acetylated N-acetylmannosamine, gangliosides, 4-*O*-acetylated glycoproteins, and glucose are not good substrates for this esterase [243]. It was shown that the *O*-acetyl esterase of ICV binds to the *N*-acetyl neuraminic acid causing a conformational change in the Ser57 of the active esterase site [244]. A study of synthetic sialic acid analog, 9-*N*-acetyl-Neu5Ac, was compared with 9-*O*-acetyl-Neu5Ac and found that ICV agglutinated both sialic acids with the same titers; however, ICV acetyl esterase could not cleave the synthetic analog [245,246]. Furthermore, it was also deduced that 9-*O*-acetyl-*N*-acetylneuraminic acid in α (2,8) linkage can also bind to ICV, along with α (2,3) and α (2,6) linkages [247]. A single point nucleotide mutation at 872 positions from C to T in the HEF protein changes the amino acid from Threonine to Isoleucine resulted in broad cell tropism by increasing the receptor binding efficiency [248].

Influenza D virus, such as ICV, utilizes the 9-*O*-acetylated sialic acids as its receptor for virus entry. HEF is the main structural protein involved in receptor binding. Although structurally similar, it was found that IDV possesses an open receptor-binding cavity compared with ICV, which enables it to bind to diverse glycan moieties. This explains the broad cell tropism exhibited by the IDV compared with ICV. IDV-HEF can also bind to both α2–3 and α2-6-linked 9-*O*-Ac-Sialic acids. IDV can also bind to the 9-*O*-acetylated sialic acid receptors which have either acetyl (Neu5,9(Ac)2 or glycolyl (Neu5Gc9Ac) moieties at the C5 position, whereas ICV shows a strong preference to Neu5,9(Ac)2 [249]. The location of the receptor-binding site of HEF is close to the top of the HEF1 globular head and forms a shallow cavity in both ICV and IDV. The receptor-binding site is flanked by the residues that form secondary structures of 170-loop, 190-loop, 230-helix, and 270-loop. The bottom of this cavity was formed by five base residues which are distinct for IDV and ICV, F127 (C/HEF: Y127), W185 (C/HEF: L184), Y231 (C/HEF: Y227), F229 (C/HEF: F225), and F297 (C/HEF: F293). A striking feature of the IDV-HEF receptor binding site is the presence of an open channel between the 230 helix and the 270 loop formed by T239 and A273, respectively, whereas in ICV-HEF, K235 of the 230 helix and D269 of the 270 loop form a salt bridge, which pulls the 270 loop close to the 230 helix, causing a close channel [75], which is a limitation for its binding efficiency and hence the reason for restricted cell/tissue tropism of ICV. 

### 11.3. 9-O-Acetyl Sialic Acids in Mammalian Tissues and Virus Tropism

The acetylated sialic acid distribution is a determinant of the susceptibility of ICV or IDV in that particular species. Using a recombinant soluble form of ICV-HE-Fc with Fc portion of human IgG1, tissue cryosections of adult Sprague Dawley rats were used to determine the 9-*O*-acetyl sialic acids, and it was found that strong, yet variable, amounts of 9-*O*-acetyl sialic acids are found in the grey matter of the brain, liver parenchymal cells, and endothelium of the central vein, luminal cells of the colon mucosa, glomeruli of the kidney, cells of the adrenal medulla, etc. The retinal layers were stained with variable intensities; however, parenchymal cells of the submaxillary gland, heart, pancreas, heart, testis, and skeletal muscle did not show any staining [250]. Among the common cell lines used for the influenza studies, 9-*O*-Ac sialic acids are seen in HEK293, A549, MDCK, and equine dermal fibroblast cells (NBL6) [251]. The sialic acid distribution, especially of the 9-*O*-acetylated sialic acids, is not well studied. However, some in vitro lectin binding assays to determine the different species of acetylated sialic acids and linkages have been performed, which is summarized in Table 6.

### 11.4. Species-Specific Host Restriction Factors of ICV and IDV

A study in 1955 demonstrated that non-specific inhibitors in the rat serum inhibited the influenza C virus, unlike sera from chickens, hamsters, ferrets, mice, rabbits, or guinea pigs [252,253]. Glycoproteins with known HA inhibitor activity such as collocalia mucoid, a glycoprotein from human and rhesus erythrocytes, fetuin, and neuramine lactose did not inhibit ICV; however, rat serum extract inhibited ICV [218]. The disruption of the interaction of the rat serum inhibitor (RSI) with hemagglutinin glycoprotein at 23–90 °C for 20–90 min was studied, and it was shown that RSI could be a receptor analog, that interacts with HA and RDE of ICV [254]. Purified α1-macroglobulin (RMG) is the active component from rat plasma that can inhibit the ICV hemagglutination [255]. Neuraminidase treatment of RMG decreased the inhibitory activity suggesting the sialic acid is the essential component in the proposed receptor for ICV [255,256]. Only the rat alpha 1 and alpha 2 macroglobulins and rat murinoglobulin I and II inhibited the hemagglutination, whereas neither of these proteins from mouse or guinea pig plasma showed inhibition [257].

The rat serum contains two ICV inhibitors, alpha 1-macroglobulin, and murinoglobulin, and these globulins inhibited ICV agglutination and not IAV and IBV. These inhibitors are specific to ICV, as the inhibitory property was abolished by ICV only and not by A and B viruses at 37 °C [258]. These inhibitors also showed susceptibility towards neuraminidase from *Arthrobacter ureafaciens*, sodium hydroxide, and methylamine and refractory to neuraminidase from *Vibrio cholerae*, and sodium metaperiodate [258]. 

Human MxA protein was also found to have inhibitory action against ICV [259]. Similar to A/WSN, C/JHB/1/66 also demonstrated a decrease in infectivity titers by actinomycin D and alpha-amanitin, which also confirmed that ICV shares properties with other orthomyxoviruses during early times [260]. Baloxivir, the RNA polymerase inhibitor, has inhibition properties against four different types of influenza viruses, and it was found that the level of inhibition in the increasing order is A > B > C > D by inhibiting the cap-dependent endonuclease activity of the PA [261]. The amino acid residue at position 38 active site of the PA or P3 protein is the key determinant of the Baloxivir susceptibility [261]. 

### 11.5. Antiviral Innate Immunity to ICV and IDV

The specific antiviral innate immunity against ICV and IDV has not been studied; hence, there is very limited information. The C-terminal portion of the NS1 protein of C/JJ/50 and C/JHB/1/66 can modulate the IFN-β promoter activity by inhibiting the RIG-I mediated signaling independent of the RNA-binding mechanism [211]. It was reported that pre-existing immunity of IDV is weak and offers limited cross-protection, as indicated by an experimental study. This limited protection as well as the presence of diverse genetic variants in co-circulation caused re-infection, and thereby a high prevalence of IDV in cattle [147]. In the case of IDV, the third (136–146 aa) nuclear export signal (NES) of IDV NS2 among the three separate NES regions is majorly responsible for the nucleocytoplasmic transport through the chromosome region maintenance 1/Xpo1 (CRM1) pathway [262]. With the increased clinical cases of ICV-related pneumonia in children along with the absence of antiviral immunity specific to ICV, more and more studies are needed in this area.

## 12. Conclusions and Future Perspectives

The intercontinental transmission and emergence of IDV in the European countries have been discussed in light of the contributing factors; similar studies are needed to study the emergence of ICV in animal species across the world. Given the long history of the existence of ICV compared with IDV, the virus is much less studied. The fact that these two seven-segmented genome viruses have several lineages, capable of undergoing recombinations and reassortments, coupled with their active co-existence in bovine and swine species, common genomic composition, and receptor specificities, could potentially enable these viruses to broaden the host range by species spillover. Furthermore, the detection of IDV in all the continents and the seroprevalence of IDV and ICV in cattle, swine, camelids, small ruminants, horses, and more importantly in humans, suggests the potential means for bidirectional transmission and public health risks.

## Figures and Tables

**Table 1:** Summary of the seroepidemiological studies of ICV conducted in humans and other animal species.

Year	Country	Species	Seropositivity		References
			Age group	Prevalence	
1978	USA	Human (*n* = 334)	0–5 years	64%	[62]
			6–10 years	96%	
			11–15 years	100%	
			16–25 years	98%	
1980	USA	Human (*n* = 237)	1–2 years	36%	[63]
			2–5 years	47.2%	
			20–30 years	96%	
			65–85 years	66.7%	
1981	China	Pigs		3%	[35]
1983	Japan	Human (*n* = 653)	2–4 years	50%	[66]
			≥5 years	90–100%	
1984	Russia	Human	Not reported		[67]
1985	Russia	Human (*n* = 975)	adults (20–60 years)	85.7%	[68]
1987	Japan	Dogs (*n* = 112)	Not reported	4.5%	[21]
1987	Japan	Pigs (*n* = 269)	Not reported	38%	[21]
1991	Japan	Pig (*n* = 240)	Not reported	19%	[36]
1992	France	Dogs (*n* = 134)	6 months–16 years	32%	[20]
1992	France	Humans (*n* = 301)	4 months–88 years	61% (High in 16–30 years group)	[69]
1993	Spain	Dogs	Not reported	56.3%	[70]
1996	Bavaria	Dogs (*n* = 150) and pigs (*n* = 240)	Not reported	Dogs—50.6% Pigs—49.9%	[71]
2000	Brazil	Humans (*n* = 67)	Not reported	56.7%	[72]
2020	Japan	Humans	21–66 years	17.5%	[64]

**Table 2:** Identification and Seroprevalence of influenza D (in percentages) in different mammalian species.

Country	Year	Bovine	Small Ruminant	Swine	Equine	Camelid	Human	Ref.
USA #	2013			9.5			* 1.3	[82]
	2016						** 94–95	[26]
	2015		S: 5.2,					[27]
			G: 8.8					
	2017				11–12			[46]
China #	2017	DC: 7.8,	G: 33.8					[45]
		B: 5.9						
France #	2014–18	47.2	1.5					[80]
Japan #	2016	30.5						[41]
Togo	2009,2015 2017–20	10.4 6.3	S: 2; G:4.4					[32,81]
Morocco	2012–15	35						[81]
Kenya	2015					99–100		[32]
Cote-d’Ivoire	2017–19	7.2	S: 4.1, G:3.7					[81]
Benin	2017–19	3.9						[81]
Uganda	2017–19	20.9		20.5				[81]
Ethiopia	2019					55.2		[42]
Italy #	2004						4.88	[93]
	2005–17						5.1–46	[23]
	2016			11.7				[78]
Ireland #	2015, 2017	94.6	4.5	5.8				[40,94]
Mexico #	2015							[87]
Luxembourg	2016	80.2						[43]
	2014–15			5.9				
Argentina	2013	68						[39]
Turkey #	2018–19							[92]
Western Canada	2018–19	7.1						[89]

* General population; ** Occupational workers; # Seroprevalence with virus isolation; DC—Dairy cattle; B—Buffalo; G—Goats; S—sheep.

**Table 3:** Genome segments of influenza C and D viruses and their encoded proteins.

Segment	Protein	hICV		bIDV		Function
		nt ^#^	aa *	nt	aa	
1	PB2	2365	774	2364	772	Cap-binding, form heterotrimeric polymerase complex for replication and mRNA synthesis
2	PB1	2363	754	2330	753	Contains Polymerase active site, form heterotrimeric polymerase complex for replication and mRNA synthesis
3	P3	2183	709	2195	710	Contains endonuclease domain for cap-snatching, form heterotrimeric polymerase complex for replication and mRNA synthesis
4	HEF	2073	655	2049	664	Spike protein, receptor binding, fusion, and receptor destroying functions
5	NP	1807	565	1775	552	Forms viral ribonucleoprotein complex (vRNPs) together with polymerases and vRNA
6	P42	1180	374	1219	387	
	M1	1125	242		246	Structural protein that lines the viral membrane inside
	M2	420	139		148	Transmembrane protein with ion proton-channel activity
7	NS	935	286	868		
	NS1	740	246	732	243	Interferon antagonist
	NS2/NEP	549	182	555	184	Nuclear export of RNPs

# nt—Nucleotide; * aa—amino acid; hICV—C/Victoria/2/2012; bIDV—D/bovine/Oklahoma/660/2013.

**Table 4:** Sequence identities of ICV and IDV as determined by BLASTn and BLASTp.

Gene	bICV vs. hICV		swICV vs. hICV		bICV vs. swICV		bIDV vs. hICV		bIDV vs. bICV		swIDV vs. swICV	
	nt	aa	nt	aa	nt	aa	nt	aa	nt	aa	nt	aa
PB2	94.5	98.97	98.8	99.87	95.10	99.1	65.07	52.59	66.45	52.59	66.28	52.72
PB1	94.25	98.67	98.62	99.34	94.86	98.81	69.69	72.44	70.15	72.84	69.48	72.57
P3	96.43	98.59	96.03	98.59	95.61	98.03	65.42	49.86	65.48	50.14	74.74	49.86
HEF	89.99	92.6	94.69	96.15	91.33	93.84	67.44	52.64	67.22	52.85	67.46	53.5
NP	95.83	97.7	96.92	99.29	95.32	97.52	85.29	39.33	85.29	39.25	77.78	39.92
M	95.99	97.06	99.38	100.0	95.95	97.06	95.65	35.94	- *	36.20	- *	37.76
NS	94.83	94.72	98.18	97.97	95.69	95.93	100	33.04	85.71	32.46	85.71	32.89

*—No significant similarity found; nt—Nucleotide; aa—amino acid; bICV—C/bovine/Montana/12/2016; hICV—C/Victoria/2/2012; swICV—C/pig/Beijing/115/81; bIDV—D/bovine/Oklahoma/660/2013; swIDV—D/swine/Oklahoma/1334/2011.

**Table 5:** Predicted glycosylation sites of different IDV lineages as predicted by NetNGlyc 1.0 server (threshold 0.5).

Viruses	Accession No.	No: of Sites	Glycosylation Sites
D/bovine/Oklahoma/660/2013	AGS48804.1	7	N(28)ES, N(54)VT, N(146)WT, N(249)KT, N(346)AT, N(513)DT, N(613)GS
D/swine/Oklahoma/1334/2011	YP_009449559.1	6	N(28)ES, N(54)VT, N(146)WT, N(346)AT, N(513)DT, N(613)GS
D/bovine/Ibaraki/7768/2016	BAV17997.1	6	N(28)ES, N(54)VT, N(146)WT, N(346)AT, N(513)DT, N(613)GS
D/bovine/Yamagata/1/2019	BBM60897.1	8	N(28)ES, N(54)VT, N(146)WT, N(249)KT, N(346)AT, N(390)DT, N(513)DT, N(613)GS
D/bovine/Califonia/0363/2019	QOF88712.1	7	N(28)ES, N(54)VT, N(146)WT, N(249)RT, N(346)AT, N(513)DT, N(613)GS

**Table 6:** Presence of 9-*O*-acetylated sialic acids and sialic acid linkages in tissues of different mammalian species based on lectin staining.

Species	Tissue		Sialic Acid			Linkages	
		9-*O*-Ac	7,9-*O*-Ac	5,9-*O*-Ac	4-*O*-Ac	α2-3-linked Sias	α2-6-linked Sias
Human	Trachea Lung	++ +++	++ ++	ND	_ _	+++ +++	+++ ++
Mouse	Trachea Lung Saliva RBC	+++ +++ ND ND	++ +++ ND ND	ND ND +++ +++	+ + ND ND	+ +++ ND ND	+ + ND ND
Ferret	Trachea Lung	_ +	_ _	ND ND	_ _	+ +	+ a +
Guinea pig	Trachea Lung	+ +	_ _	ND ND	+++ b +++	++ ++	+ ++
Pig	Trachea Lung	+ _	++ _	ND ND	_ _	+++ +++	+++ +++
Horse	Trachea Lung Saliva RBC	++ + ND ND	+ - ND ND	ND ND +++ _	+++ +++ ND ND	+++ +++ ND ND	+++ +++ ND ND
Dog	Trachea Lung	+++ _	+++ _	ND ND	_ _	+++ ++	++ +++
Cow	Saliva RBC	ND ND	ND ND	+++ _	+++ ND	ND ND	ND ND

+++: Good amount; ++: Little; +: Limited; _: Not present; ND: Not determined. ^a^ present in the submucosal glands; ^b^ Not in the epithelium, present in the submucosa. Refs: [250,251].

## Data Availability

Not applicable.
